# *Foeniculum vulgare* Mill: A Review of Its Botany, Phytochemistry, Pharmacology, Contemporary Application, and Toxicology

**DOI:** 10.1155/2014/842674

**Published:** 2014-08-03

**Authors:** Shamkant B. Badgujar, Vainav V. Patel, Atmaram H. Bandivdekar

**Affiliations:** Department of Biochemistry, National Institute for Research in Reproductive Health, ICMR, Jehangir Merwanji Street, Parel, Mumbai, Maharashtra 400 012, India

## Abstract

*Foeniculum vulgare* Mill commonly called fennel has been used in traditional medicine for a wide range of ailments related to digestive, endocrine, reproductive, and respiratory systems. Additionally, it is also used as a galactagogue agent for lactating mothers. The review aims to gather the fragmented information available in the literature regarding morphology, ethnomedicinal applications, phytochemistry, pharmacology, and toxicology of* Foeniculum vulgare*. It also compiles available scientific evidence for the ethnobotanical claims and to identify gaps required to be filled by future research. Findings based on their traditional uses and scientific evaluation indicates that* Foeniculum vulgare* remains to be the most widely used herbal plant. It has been used for more than forty types of disorders. Phytochemical studies have shown the presence of numerous valuable compounds, such as volatile compounds, flavonoids, phenolic compounds, fatty acids, and amino acids. Compiled data indicate their efficacy in several* in vitro *and* in vivo *pharmacological properties such as antimicrobial, antiviral, anti-inflammatory, antimutagenic, antinociceptive, antipyretic, antispasmodic, antithrombotic, apoptotic, cardiovascular, chemomodulatory, antitumor, hepatoprotective, hypoglycemic, hypolipidemic, and memory enhancing property.* Foeniculum vulgare* has emerged as a good source of traditional medicine and it provides a noteworthy basis in pharmaceutical biology for the development/formulation of new drugs and future clinical uses.

## 1. Introduction

*Foeniculum vulgare* is the oldest valid name within the genus* Foeniculum* for the plant designated by Karsten as* Foeniculum Foeniculutn*. However, according to the international rules of nomenclature, the binomial name* Foeniculum vulgare* was not validly published by Hill in his reference [1] for the reason that he did not consistently adopt the binomial system of nomenclature. In accordance with the international rules as adopted at Cambridge, the name* Foeniculum vulgare* must be accredited to Philip Miller, who first validly published it in the eighth edition of his “Gardeners Dictionary” in 1768. From then on, the name of this plant is written as* Foeniculum vulgare* Mill. It is a medicinal plant belonging to the Umbelliferae (Apiaceae) family, known and used by humans since antiquity, due to its flavor. It was cultivated in almost every country [2]. It is universally known as* Fennel *and is known by more than 100 names (Table 1). It is a traditional and popular herb with a long history of use as a medicine. A series of studies showed that* F. vulgare *effectively controls numerous infectious disorders of bacterial, fungal, viral, mycobacterium, and protozoal origin [3–7]. It has antioxidant, antitumor, chemopreventive, cytoprotective, hepatoprotective, hypoglycemic, and oestrogenic activities [8–12]. Some of the publications stated that* F. vulgare* has a special kind of memory-enhancing effect and can reduce stress [13]. Animal experiments and limited clinical trials suggest that chronic use of* F. vulgare* is not harmful. Fennel maybe consumed daily, in the raw form as salads and snacks, stewed, boiled, grilled, or baked in several dishes and even used in the preparation of herbal teas or spirits. A diet with desired quantity of fennel could bring potential health benefits due to its valuable nutritional composition with respect to presence of essential fatty acids [14]. In recent years, increased interests in improvement of agricultural yield of fennel due to its medicinal properties and essential oil content has encouraged cultivation of the plant on large scale.

**Figure 1:**
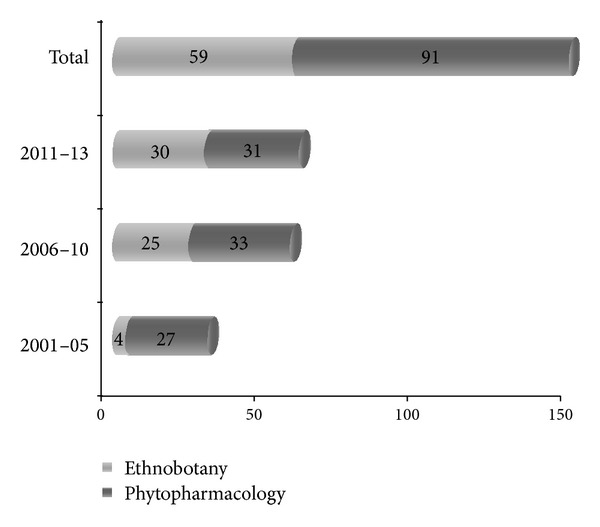
Research papers in different aspects especially traditional or ethnobotanical knowledge, phytochemistry, pharmacological, and various biological activities of* Foeniculum vulgare*. (Papers were collected via electronic databases such as Academic Journals, Ethnobotany, Google Scholar, PubMed, and Science Direct.)

Research on* F. vulgare *with current technology has been conducted all over the world. All the available literature on* F. vulgare *was compiled from electronic databases such as Academic Journals (including high impact, nonimpact, and nonindexed journals), Ethnobotany, Google Scholar, Scopus link, PubMed, Science Direct, Web of Science, and library search. A review of the literature from 2001 to 2005 shows only 20% reports published on* F. vulgare* which increased to about 38% from 2006 to 2010. Briefly, in these 10 years a total of 89 claims appeared in the literature on various aspects of* F. vulgare*. It is important to note that about 39% of reports (61 articles) were collected from recent three years, that is, 2011 to 2013 (Figure 1). Some of the earlier published reviews of this plant included medicinal properties and phytochemistry [15–20], but few of them appear in all these reviews. However, there is a need for an inclusive review that bridges the gaps between traditional uses of fennel and its* in vitro* studies. The present review attempts to collate the available information on the botany, nation-wise common vernacular names, cultivation (propagation), nutritive value, and traditional/contemporary as well as allied applications, phytochemistry, pharmacology, and toxicity of* F. vulgare*. We hope that this review may provide scientific basis that explains the ethnophytopharmacological role of* F. vulgare* in order to facilitate and guide future research. In particular, we aimed to answer the following questions. (1) What information is available on the traditional uses, botany, phytochemistry, and toxicity of* F. vulgare*? (2) What pharmacological studies were performed on this plant and how do they validate its traditional uses? (3) What is the future for* F. vulgare*?

### 1.1. Taxonomy

*Kingdom*: Plantae,* division*: Tracheophyta,* subdivision*: Spermatophytina,* class*: Magnoliopsida,* order*: Apiales,* family*: Apiaceae,* genus*:* Foeniculum*,* species*:* vulgare*, and* botanical name*:* Foeniculum vulgare* Mill.

### 1.2. Botanical Description

Fennel is an ancient seasonal herb. The fennel plant originated in the southern Mediterranean region and through naturalization and cultivation it grows wild throughout the Northern, Eastern, and Western hemispheres, specifically in Asia, North America, and Europe. It is cultivated in fields and also grows wild. The herb was well-known to the ancient Egyptians, Romans, Indians, and Chinese. The Romans grew it for its aromatic seeds and the edible fleshy shoots are still a very common vegetable in southern Italy [21]. Emperor Charlemagne was known to have encouraged its cultivation in Central Europe. It is an indispensable ingredient in modern French and Italian cooking. All parts of the plant are aromatic and can be used in many ways.

**Figure 2:**
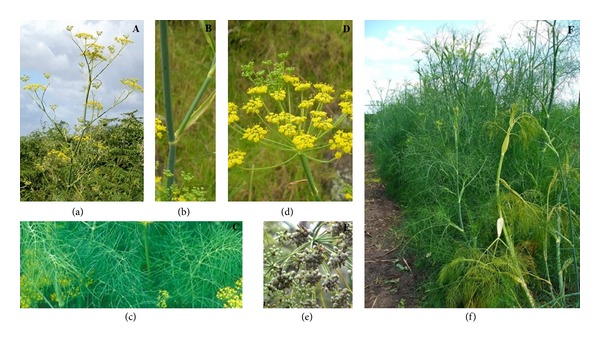
*Foeniculum vulgare* Mill (a) in its natural habitat; (b) stem; (c) leaves; (d) inflorescences and flowers; (e) fruits; and (f) population of* F. vulgare* Mill.

*F. vulgare* is an upright, branching perennial herb (Figure 2(a)) with soft, feathery, almost hair-like foliage growing upto 6.6 ft. (2 m) tall. This plant looks similar to dill. It is typically grown in vegetable and herb gardens (Figure 2(f)) for its anise-flavored foliage and seeds, both of which are commonly harvested for use in cooking. It is erect and cylindrical, bright green, and smooth as to seem polished, with multiple branched leaves (Figure 2(c)) cut into the finest of segments. The leaves grow upto 40 cm long; they are finely dissected, with the ultimate segments filiform (threadlike), about 0.5 mm wide. The bright golden flowers, produced in large, flat terminal umbels, with thirteen to twenty rays, bloom in July and August (Figure 2(d)).

**Figure 3:**
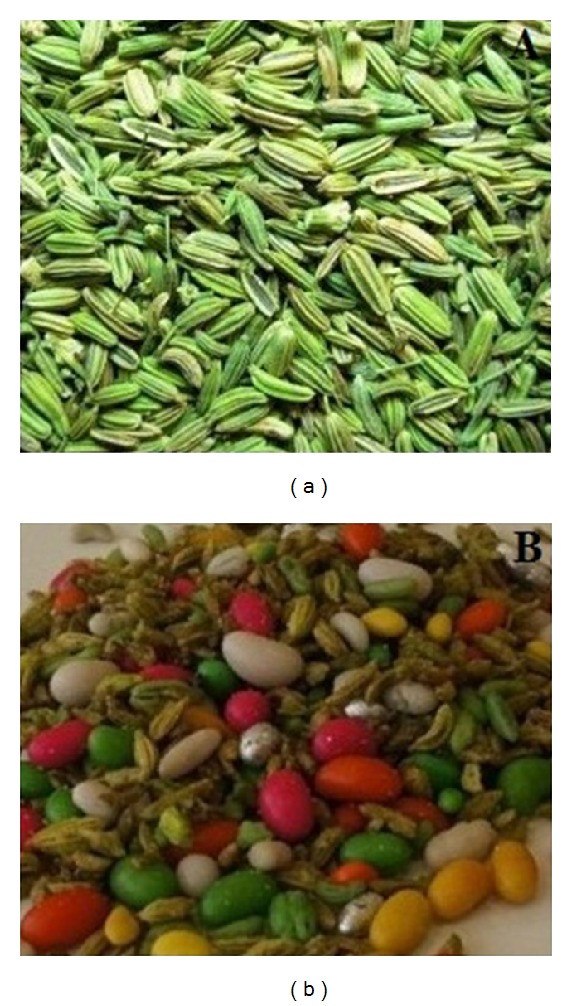
Normal fennel seeds (a) and sugar coated and uncoated fennel seeds (b) used in* mukhwas.*

*Foliage.* Stem striate, leaves 3-4 pinnate, segments filiform, upto 1.6 in. (4 cm) long; leaf bases sheathing. It has a green, sleek, and slippery stem with upright stiff branches and much divided leaves in linear segments (Figure 2(b)). Rays are 5–30 numbers with 0.39–2.4 inches (1–6 cm) long. Flowers are small, yellow, and found in large flat-topped umbels (Figure 2(d)). Fruits are oblong to ovoid with 0.12–0.2 inches (3–5 mm) long and 1.5–2.0 mm broad (Figure 2(e)). The stylopodium persists on the fruit. The fruits are elongated and have strong ribs. The most esteemed fennel seeds vary from three to five lines in length and are elliptical, slightly curved, and somewhat obtuse at the ends (Figure 3(a)). They are greenish-yellow, the colour of hay, from which the term fennel is derived. Wild fruits are short, dark coloured and blunt at their ends, and have a less agreeable flavour and odour than those of sweet fennel. Seeds ripen from September to October. This plant can reproduce from crown or root fragments but freely reproduces from seed.

### 1.3. Chemical Composition and Nutritional Value of Fennel

*Foeniculum vulgare* is widely grown for its edible fruit or seeds. These are sweet and dry; a fully ripe specimen is an exquisite fruit. The fruit is often dried for later use and this dried fruit called fennel is a major item of commerce.  Table 2 lists the nutrient composition of fennel (*USDA data*). Fennels are one of the highest plant sources of potassium, sodium, phosphorus, and calcium. According to USDA data for the Mission variety, fennels are richest in dietary fiber and vitamins, relative to human needs. They have smaller amounts of many other nutrients.

Table 3 summarizes the chemical composition and the nutritional value [14] of different parts of fennel, namely, shoots, leaves, stems, and inflorescence. Leaves and stems show the highest moisture content (76.36 and 77.46 g/100 g, resp.), while inflorescence exhibits the lowest content (71.31 g/100 g). Carbohydrates are the most abundant macronutrients in all the parts and range from 18.44 to 22.82 g/100 g. Proteins, reducing sugars, and fats are the less abundant macronutrients; proteins varied between 1.08 g/100 g in stems and 1.37 g/100 g in inflorescences. The inflorescences and stems revealed the highest fat content (1.28 g/100 g) and reducing sugar content (1.49 g/100 g), respectively, amongst all the parts of fennel. On the basis of the proximate analysis, it can be calculated that a fresh portion of 100 g of these parts yields, on average, 94 Kcal of energy. The highest values were obtained for inflorescences, while leaves and stems gave the lowest energy contribution.

About twenty-one fatty acids were identified and quantified from the above mentioned parts of fennel (Table 3). These are caproic acid, caprylic acid, capric acid, undecanoic acid, lauric acid, myristic acid, myristoleic acid, pentadecanoic acid, palmitic acid, heptadecanoic acid, stearic acid, oleic acid, linoleic acid, *α*-linolenic acid, arachidic acid, eicosanoic acid, cis-11,14-eicosadienoic acid, cis-11,14,17-eicosatrienoic acid + heneicosanoic acid, behenic acid, tricosanoic acid, and lignoceric acid. Thus, Barros and his coworker conclude polyunsaturated fatty acids (PUFA) to be the main group of fatty acids present in all the fennel parts. On the other hand Vardavas and his coworker reported monounsaturated fatty acids (MUFA) as the main group of fatty acids in fennel [22]. Nevertheless, unsaturated fatty acids (UFA) range from 66% to 80% and predominate over saturated fatty acids [14]. The highest concentration of n-3 fatty acids was found in fennel leaves, while the lowest concentration was found in inflorescences. The ratio of *ω*6 to *ω*3 fatty acids has an important role in the human diet. The highest levels of n-3 fatty acids found in leaves contributed to its lowest ratio of *ω*6 to *ω*3 fatty acids. The lowest levels of n-3 fatty acids found in inflorescences contributed to its highest ratio of *ω*6 to *ω*3 fatty acids.

Fennels have smaller amounts of many other nutrients. On a weight basis, fennels contain more calcium (49 mg/100 g) as compared with apples (7.14 mg/100 g), bananas (3.88 mg/100 g), dates (25.0 mg/100 g), grapes (10.86 mg/100 g), orange (40.25 mg/100 g), prunes (18.0 mg/100 g), raisins (40.0 mg/100 g), and strawberries (14.01 mg/100 g). Phenolics are an important constituent of fruit quality because of their contribution to the taste, colour, and nutritional properties of fruit. Amongst the phenolics analyzed in the fruit of this plant are neochlorogenic acid (1.40%), chlorogenic acid (2.98%), gallic acid (0.169%), chlorogenic acid (6.873%), caffeic acid (2.960%),* p*-coumaric acid (4.325%), ferulic acid-7-*o*-glucoside (5.223%), quercetin-7-*o*-glucoside (3.219%), ferulic acid (3.555%), 1,5 dicaffeoylquinic acid (4.095%), hesperidin (0.203%), cinnamic acid (0.131%), rosmarinic acid (14.998%), quercetin (17.097%), and apigenin (12.558%) [23].

Thus, as a typical, seasonal fresh fruit, fennels are an important constituent of the regional diet of Europe and other regions. Different varieties of fennel parts are widely used in many of the cooking dishes all over world (Table 4). Shoots, tender leaves, and stems are chewed and sucked due to their exquisite aniseed flavor. All these parts are also commonly used as vegetables. They are added raw to salads, stewed with beans and chickpeas, used to stuff fish for grilling, and placed in soups and bread bouillons. Besides seasoning, fennel is used to preserve food. Flowering stems, sugar, and honey macerating in brandy produce a highly valorized spirit. Herbal teas prepared with fresh tender or dried flowering stems are consumed chilled or hot, depending on the season.* F. vulgare* is famous for its essential oil. The characteristic anise odour of* F. vulgare, *which is due to its essential oil, makes it an excellent flavoring agent in baked goods, meat and fish dishes, ice-cream, and alcoholic beverages. The culinary uses of fennel are so diverse/widespread that it has been exported from country to country for centuries [14].

## 2. Traditional and Contemporary Uses

*Foeniculum vulgare *has been extensively used in traditional medicine for a wide range of ailments. Fennel is used in various traditional systems of medicine like in the Ayurveda, Unani, Siddha, in the Indian, and Iranian traditional systems of alternative and balancing medicine [20]. Its stem, fruit, leaves, seeds, and whole plant itself are medicinally used in different forms in the treatment of a variety of diseased conditions. The preparation methods, uses, and application of* F. vulgare *are well documented in the common ethnobotanical literature [24–32].  Table 5 lists the ethnomedicinal uses of* F. vulgare* for 43 different types of ailments in Bolivia, Brazil, Ecuador, Ethiopia, India, Iran, Italy, Jordan, Mexico, Pakistan, Portugal, Serbia, South Africa, Spain, Turkey, and USA [28, 29, 33–44]. It is used to treat simple ailments (e.g., cough/cold, cuts) to very complicated ailments (e.g., kidney ailments, cancer). It also has a wide range of veterinary uses ([45, 46] see Table  4).* F. vulgare* is used in many parts of the world for the treatment of a number of diseases, for example, abdominal pains, antiemetic, aperitif, arthritis, cancer, colic in children, conjunctivitis, constipation, depurative, diarrhea, dieresis, emmenagogue, fever, flatulence, gastralgia, gastritis, insomnia, irritable colon, kidney ailments, laxative, leucorrhoea, liver pain, mouth ulcer, and stomachache (Table 5).

In addition to its medicinal uses, aerial parts, namely, leaf, stem, and fruit/seed of* F. vulgare,* are extensively used as galactagogues not only for increasing the quantity and quality of milk but also for improving the milk flow of breastfeeding mothers [32, 34, 37, 47]. From ancient times, fennel seeds have been used as an ingredient for removing any foul smell of the mouth [48]. The natural light green dye obtained from leaves is used in cosmetics, for coloring of textiles/wooden materials and as food colorant. Yellow and brown color dyes are obtained by combining the flowers and leaves of fennel [49]. In Portugal, Italy, Spain, and India, the stem, fruit, leaves, seeds, and whole plant are used as a vegetable [3, 9, 48, 50, 51]. Sugar coated and uncoated fennel seeds are used in* mukhwas *(Mouth freshener) (Figure 3(b)). In many parts of India and Pakistan, roasted fennel seeds are consumed as* mukhwas *(Mouth freshener).* Mukhwa*s is a colorful after-meal mouth freshener or digestive aid. It can be made of various seeds and nuts but often found with fennel seeds, anise seeds, coconut, and sesame seeds. They are sweet in flavor and highly aromatic due to the presence of sugar and the addition of various essential oils. The seeds can be savory, coated in sugar, and brightly colored.

## 3. Phytochemistry

**Figure 4:**
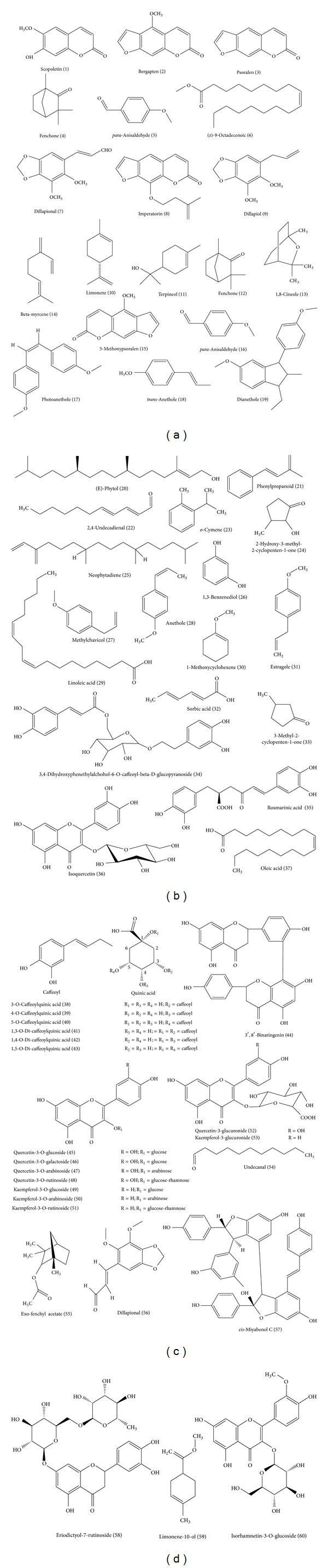
Chemical structures of various phytoconstituents isolated from* Foeniculum vulgare.*

Phytochemical research carried out on* Foeniculum vulgare *has led to the isolation of fatty acids, phenolic components, hydrocarbons, volatile components, and few other classes of secondary metabolites from its different parts (Figure 4). Mostly these phytochemicals are found in essential oil (Table 6). Some of the phytoconstituents of* F. vulgare *were find application as coloring and antiaging agents [49, 50]. They also have noteworthy biological and pharmacological activities (Table 7).

### 3.1. Volatile Compounds

Table 6 summarizes the volatile compounds present in the essential oil of* F. vulgare*. The anise odor of* F. vulgare* is due to its essential oil content. It makes an excellent flavoring agent in various types of food and food related products. The essential oil of fennel has been reported to contain more than 87 volatile compounds [51–57]. The accumulation of these volatile compounds inside the plant is variable, appearing practically in any of its parts, namely, roots, stem, shoots, flowers, and fruits [58, 59]. The molecular structures of major volatile components of* F. vulgare* seed essential oil have been illustrated in Figure 4.

Guillén and Manzanos [60] investigated the yield and composition of the volatile components found in the pentane extracts of leaves, stems, and seeds of* F. vulgare*. They identified a total of 37 volatile compounds from pentane extracts of above mentioned parts of fennel by using gas chromatography (GC) and gas chromatography-mass spectrometry (GC-MS) techniques. In the supercritical CO_2_ (SC-CO_2_) seed extracts of fennel, a total of 28 compounds were identified with major compounds being* trans*-anethole (68.6–75.0%), fenchone (8.40–14.7%), and methylchavicol (5.09–9.10%) whereas only 19 compounds were detected from hydrodistilled oil of fennel [52]. Fang et al. [53] characterizes 76 volatile components in the essential oil of* F. vulgare* with the help of three advanced techniques, namely, headspace solvent microextraction followed by gas chromatography-mass spectrometry (HSME-GC-MS), solid phase microextraction- (SPME-) GC-MS, and steam distillation- (SD-) GC-MS methods. In 2007 Tognolini et al*. investigated* the chemical composition of essential oil of fennel. GC/MS study revealed a total of 18 compounds present in it with anethole being the most abundant [55]. A comparative profile of occurrence of monoterpene hydrocarbons, oxygenated monoterpenes, and phenylpropanoids with respect to various maturity stages (immature, premature, mature, and fully mature) of the fruit of* F. vulgare *was reported by Telci et al. [56]. They concluded that the content of essential oil decreases with increasing maturity. A total of 28 components of the essential oil were identified, accounting for 98.0% of the total oil. The principal compound in the essential oil was* trans*-anethole (72.2%) followed by estragole (7.6%), d-limonene (6.8%), and fenchone, that is, 3.9% [61]. Overall, 60 compounds representing 90.1–98.7% of the essential oil were identified by GC and GC/MS in the two cultivars of fennel, namely, Aurelio and Sparta cocultivars. The major constituent of the essential oils is* trans*-anethole (59.8–90.4%). In addition, the fennel essential oils also contains minor amounts of various constituents as limonene (0.1–21.5%), neophytadiene (0–10.6%), (E)-phytol (0.1–6.0%), exo-fenchyl acetate (0.3–3.8%), estragole (0.1–2.5%), and fenchone, that is, 0.1–3.1% [62]. In addition, Zoubiri et al. [57] summarized the comparative profile of volatile compounds found in different varieties of fennel from different countries such as Estonia, Norway, Austria, Moldova, and Turkey. The chemical composition of the Algerian* F. vulgare* seed oil was different as compared with Turkish [51, 56], Serbian [52], Indian [54], and Chinese [53] fennels. The hexane extracts of fennel were analyzed by GC-MS and 78 compounds were identified from these extracts; the major compounds were identified as 1,3-benzenediol, 1-methoxycyclohexene,* o*-cymene, sorbic acid, 2-hydroxy-3-methyl-2-cyclopenten-1-one, estragole, limonene-10-ol, and 3-methyl-2-cyclopenten-1-one [63]. Diao et al. [64] identify a total of 28 components by GC and GC/MS from fennel oil, representing 95.8% of the total amount.* Trans*-Anethole (68.53%), a phenylpropanoid, was found to be the main component, followed by estragole (10.42%) with limonene (6.24%), fenchone (5.45%), and others as minor components.

### 3.2. Flavonoids

Flavonoids are generally considered as an important category of antioxidants in the human diet. Flavonoids are abundant in the plants of Apiaceae family. It has been reported that the presence of flavonol glycosides in fennel species is related to its morphological heterogeneity and variation. Total flavonoid content of hydroalcoholic extracts is about 12.3 ± 0.18 mg/g. Flavonoids like eriodictyol-7-rutinoside, quercetin-3-rutinoside, and rosmarinic acid have been isolated from* F. vulgare* [65]. Amongst the flavonoids present in* F. vulgare*, the most prevalent are quercetin-3-glucuronide, isoquercitrin, quercetin-3-arabinoside, kaempferol-3-glucuronide and kaempferol-3-arabinoside, and isorhamnetin glucoside [66]. Quercetin-3-*O*-galactoside, kaempferol-3-*O*-rutinoside, and kaempferol-3-*O*-glucoside have also been reported to occur in the aqueous extract of* F. vulgare* [67]. The flavonoids like isorhamnetin 3-*O*-*α*-rhamnoside, quercetin, and kaempferol were also isolated from the ethyl acetate extract, whereas quercetin 3-*O*-rutinoside, kaempferol 3-*O*-rutinoside, and quercetin 3-*O*-*β*-glucoside were isolated from the methanol extract. These flavonoids exhibit remarkable antinociceptive and anti-inflammatory activity [68]. Further, quercetin, rutin, and isoquercitrin were reported to have the immunomodulatory activities [69].

### 3.3. Phenolic Compounds

There has been a growing interest in phenolic components of fruits and vegetables, which may promote human health or lower the risk of disease. Aqueous extract of fennel fruits are rich in phenolic compounds. Many of them have antioxidant activities and hepatoprotective properties. The phenolic compounds present in* F. vulgare* are considered to be associated with the prevention of diseases possibly induced by oxidative stress such as cardiovascular diseases, cancer, and inflammation. These phenolic compounds have received tremendous attention among nutritionists, food scientists, and consumers due to their role in human health. Fennel has been reported to contain hydroxyl cinnamic acid derivatives, flavonoid glycosides, and flavonoid aglycones [67]. The methanolic extract of fennel seeds contains rosmarinic acid, chlorogenic acids as major phenolic compounds (14.9% and 6.8%, resp.), and quercetin and apigenin as the major flavonoids (17.1% and 12.5%, resp.). Also, the total phenolic compounds in fennel methanol extract were higher than the flavonoid compounds [23].* F. vulgare* has been reported to contain phenolic acids like 3-*O*-caffeoylquinic acid, 4-*O*-caffeoylquinic acid, 5-*O*-caffeoylquinic acid, 1,3-*O*-di-caffeoylquinic acid, 1,4-*O*-di-caffeoylquinic acid, and 1,5-*O*-di-caffeoylquinic acid [65]. Two compounds A and B were isolated and characterized for the first time from the wild fennel and identified as 3,4-dihydroxyphenethylalchohol-6-*O*-caffeoyl-*β*-D-glucopyranoside and 3′,8′-binaringenin, respectively. The total phenolic and flavonoid contents of wild fennel (2.4% and 1.2% resp.) were less as compared to cultivated fennel (3.1% and 1.6%, resp.) [70].

## 4. Pharmacological Activities

*Foeniculum vulgare* is officially noted in* Ayurvedic* Pharmacopoeia as an important part of polyherbal formulations in the treatment of different diseases and disorders. A number of biological-pharmacological studies have been undertaken to evaluate the indigenous uses of* F. vulgare*. Few extracts of* F. vulgare *and isolated compounds have been evaluated for several activities, namely, antiaging, antiallergic, anticolitic, antihirsutism, anti-inflammatory, antimicrobial and antiviral, antimutagenic, antinociceptive, antipyretic, antispasmodic, antistress, antithrombotic, anxiolytic, apoptotic, cardiovascular, chemomodulatory action, cytoprotection and antitumor, cytotoxicity, diuretic, estrogenic properties, expectorant, galactogenic, gastrointestinal effect, hepatoprotective, human liver cytochrome P450 3A4 inhibitory, hypoglycemic, hypolipidemic, memory-enhancing property, nootropic, and oculohypotensive activities [11, 13, 20, 50, 68, 71–90].  Table 8 summarizes the pharmacological studies undertaken on* F. vulgare *and reported in the literature. A brief review of the same is as follows.

### 4.1. Antimicrobial and Antiviral Activities

*Foeniculum vulgare *has been used as an ethnic remedy for the cure of numerous infectious disorders of bacterial, fungal, viral, and mycobacterial origin. Several studies have been carried out in the past validating its antimicrobial, antimycobacterial,, and antiviral potential (summarized in the Table 9). Duško et al. [91] investigated the antibacterial effect of the aqueous extract of 12 medicinal plants of* Apiaceae* family including* F. vulgare.* An aqueous extract of the aerial part of* F. vulgare *inhibited the growth of* Agrobacterium radiobacter pv. tumefaciens*,* Erwinia carotovora*,* Pseudomonas fluorescens, *and* Pseudomonas glycinea *(Table 9). An aqueous extract of seed sample inhibited the growth of* Enterococcus faecalis, Staphylococcus aureus, Escherichia coli, Klebsiella pneumonia, Pseudomona aeruginosa, Salmonella typhi, Salmonella typhimurium, Shigella flexneri, *and* Bacillus cereus *with 13–22, 22–24, 14–24, 20-21, 21–24, 11-12, 14–18, 17–18, and 24–26 mm zone of inhibition, respectively [3, 4]. Gulfraz et al. [92] investigated the antibacterial effect of the essential oil as well as ethanolic and methanolic fruit extracts of* F. vulgare *against* Bacillus cereus*,* Bacillus megaterium*,* Bacillus pumilus*,* Bacillus subtilis*,* Escherichia coli*,* Klebsiella pneumonia*,* Micrococcus luteus*,* Pseudomonas putida*,* Pseudomonas syringae,* and* Candida albicans*. According to the results reported by Gulfraz et al. [92], essential oil of* F. vulage *had significant antimicrobial activities against some of microorganisms as compared to the methanolic and ethanolic extracts. The diameters of growth inhibition zone ranged from 14 to 31 mm (including the diameter of the disc 6 mm) with the highest inhibition zone values observed against* Bacillus megaterium *(31 mm) and* Bacillus subtilis *(29 mm). Roby et al. [23] investigated antimicrobial effect of the methanol, ethanol, diethyl ether, and hexane extracts of seed of* F. vulgare* against two species of Gram negative bacteria (*Escherichia coli *and* Salmonella typhi*), two species of Gram positive bacteria (*Bacillus cereus *and* Staphylococcus aureus*), one species of yeast (*Candida albicans*), and one species of mold (*Aspergillus flavus*). The methanolic extract showed more effective antimicrobial activity than the other extracts. The results from the disc diffusion method, followed by measurement of minimum inhibitory concentration (MIC), indicated that* Bacillus cereus *and* Aspergillus flavus *were the most sensitive microorganisms tested, showing the largest inhibition zones and the lowest MIC values. Least activity was exhibited against* Escherichia coli*, with the smallest inhibition zones and the highest MIC value [23]. Shrivastava and Bhargava [93] investigated the antibacterial effect of the crude, chloroform, and methanol extract of leaves and flowers of* F. vulgare *along with* Raphanus sativus *and* Brassica nigrum *against* Escherichia coli *and* Staphylococcus aureus*. Methanol extract of flower of* F. vulgare *showed significant activity against* Escherichia coli, *whereas crude and chloroform extracts failed to exhibit antimicrobial activity against* Staphylococcus aureus *(Table 9). Among different tested bacterial strains, the methanolic fruit extract of* F. vulgare *inhibited the growth of* Staphylococcus aureus *and* Bacillus pumilus* with 11.27 and 12.67 mm zone of inhibition, respectively [7].

Several studies indicating the antifungal effect of* F. vulgare *along with antibacterial effect are also reported in the literature. Martins et al. [94] investigated the antibacterial and antifungal effects of three essential oils of Portuguese plants, namely,* Foeniculum vulgare*,* Mentha spicata, *and* Rosmarinus officinalis *against* Staphylococcus aureus*,* Escherichia coli*,* Klebsiella pneumonia*,* Pseudomona aeruginosa*,* Staphylococcus epidermidis, Candida albicans,* and phytopathogenic molds,* Aspergillus niger *and* Fusarium oxysporum. *Essential oil of* F. vulgare *showed significant antifungal activity against the food spoilage fungi* Aspergillus niger *and* Fusarium oxysporum *and may have important applications as food additives. The MIC values of* F. vulgare *essential oil were 250 *μ*g/mL for* Fusarium oxysporum *and 750 *μ*g/mL for* Aspergillus niger *[94]. The oils extracted from* F. vulgare *exhibit varying levels of antifungal effects on the experimental mycelial growth of* Alternaria alternata*,* Fusarium oxysporum*, and* Rhizoctonia solani *[95]. Essential oil of* F. vulgare *showed appreciable antifungal activity against strains of pathogenic fungi, namely,* Aspergillus niger*,* Fusarium solani, *and* Rhizopus solani *[96]. Dichloromethane extracts and essential oils from* F. vulgare *showed antifungal activity against* Candida albicans*. It could be a potential candidate for a new antifungal agent for candidiasis and other fungal diseases [97]. In an* in vitro *study, aqueous and alcoholic seed extracts of* F. vulgare *exhibited inhibitory effect against* Alternaria alternata, Mucor rouxii, *and* Aspergillus flavus *[98]. Interestingly, aqueous seed extract of* F. vulgare *showed strongest antifungal activity as compared to reference fungicidal agent, that is, griseofulvin [99].

All of the above mentioned studies were carried out on the crude extracts and it is difficult to pinpoint the active antimicrobial metabolite. A phenylpropanoid derivative called dillapional, characterized from* F. vulgare *stem, was found to be an antimicrobial constituent with MIC values of 125, 250, and 125 against* Bacillus subtilis*,* Aspergillus niger,* and* Cladosporium cladosporioides,* respectively. A coumarin derivative, scopoletin, was also isolated as a marginally antimicrobial agent [100]. The characterization of seven different types of oxygenated monoterpenes, from methylene chloride crude extract of* F. vulgare* [101], suggested that the crude extract containing monoterpenes could be a new medicinal resource for antibacterial agents.

A total of 78 compounds were identified from the active antimycobacterial fraction of* F. vulgare* with the help of gas chromatography-mass spectra (GC-MS). Out of these, twenty compounds were tested against one sensitive and three MDR strains of* Mycobacterium tuberculosis *using the Alamar Blue microassay. Compounds that showed some degree of antimycobacterial activity against all strains tested were the following: linoleic acid (MIC 100 *μ*g/mL), oleic acid (MIC 100 *μ*g/mL), 1,3-benzenediol (MIC 100–200 *μ*g/mL), undecanal (MIC 50–200 *μ*g/mL), and 2,4-undecadienal (MIC 25–50 *μ*g/mL). 2,4-Undecadienal was the most active compound against multidrug resistant* M. tuberculosis *species. Thus, the dietary intake of* F. vulgare* may lower the risk of* M. tuberculosis *infection [63].

Orhan et al. [5] studied the antiviral activity of the essential oil of fruit sample of* F. vulgare *along with 12 other Turkish medicinal plants against the DNA virus* Herpes simplex *type-1 (HSV-1) and the RNA virus parainfluenza type-3 (PI-3). Most of the oils and compounds displayed strong antiviral effects against* HSV-1*, ranging between 0.8 and 0.025 *μ*g/mL. However, the samples tested were less effective against PI-3, with results ranging between 1.6 and 0.2 *μ*g/mL. Only the essential oils of* Anethum graveolens*,* Foeniculum vulgare *(fully mature),* Mentha piperita*,* Mentha spicata*,* Ocimum minutiflorum*,* Ocimum vulgaris*, and* Satureja cuneifolia *inhibited this virus significantly.

All these literature findings validated the traditional uses of* Foeniculum vulgare *in infectious disorders like abdominal pains, antiemetic, arthritis, conjunctivitis, constipation, depurative, diarrhea, dieresis, fever, flatulence, gastralgia, gastritis, insomnia, irritable colon, mouth ulcer, stomachache, respiratory disorders, skin diseases, and so forth. There is always a need for new antimicrobial agents due to rapid development of resistance. Bioactive metabolites of* F. vulgare *may be a potential source for new antimicrobial agents.

### 4.2. Anti-Inflammatory Activity

Oral administration of methanol extract of* F. vulgare* fruit to rat and mice exhibited inhibitory effects against acute and subacute inflammatory diseases. The anti-inflammatory activity of methanol extract was evaluated by using three screening protocols, namely, carrageenan-induced paw edema, arachidonic acid-induced ear edema, and formaldehyde-induced arthritis. These are widely used for testing nonsteroidal anti-inflammatory drugs. For acute inflammation, methanol extract (200 mg/kg) exhibits significant inhibition of paw edema (69%) induced by carrageenan injection as compared to the control group of animals. Methanol extract of* F. vulgare* also inhibits ear-edema (70%) induced by arachidonic acid in mice. The level of serum transaminase, aspartate aminotransferase (AST), and alanine aminotransferase (ALT) significantly increases in the presence of methanolic extract of* F. vulgare *on inflammation induced by formaldehyde as compared to control group. The assessment of the level of AST and ALT provides a good and simple tool to measure the anti-inflammatory activity of the target compounds [102]. These overall results seem to suggest that* F. vulgare* FME may act on both the cyclooxygenase and lipoxygenase pathways [79].

### 4.3. Antiallergic Activity

Methanolic extract of* F. vulgare* fruit showed significant inhibitory effect on DNFB- (2,4-dinitrofluorobenzene-) induced delayed type hypersensitivity after oral administration of 200 mg/kg once a day for 7 days. The inhibitory effect on immunologically induced swelling suggests the possible immunosuppressive properties of* F. vulgare* [79].

### 4.4. Hepatoprotective Activity

Essential oil of* F. vulgare* seeds revealed a potent hepatoprotective effect against acute hepatotoxicity produced by carbon tetrachloride in rats. Oral administration of* F. vulgare* essential oil decreases the levels of serum aspartate aminotransferase (AST), alanine aminotransferase (ALT), alkaline phosphatase (ALP), and bilirubin as compared to the control group. Ozbek et al. suggest that the constituents (*d*-limonene and *β*-myrcene) of essential oil may have played a key role in the protection of liver from CCl_4_ toxicity [9].

### 4.5. Anxiolytic Activity

Anxiety is the unpleasant feeling of fear and concern. When anxiety becomes excessive, it may be considered as an anxiety disorder. Anxiolytic fennel is a drug used for the treatment of anxiety and its related psychological and physical symptoms. Naga Kishore et al. [89] investigated the anxiolytic activity of ethanolic extract of* F. vulgare *fruit with the help of elevated plus maze, rota rod, open field test, and whole board models. The 100 to 200 mg dose of extract per kg of body weight of animal revealed significant activity when compared to reference anxiolytic drug called diazepam (1 mg/kg). Thus, fennel extract may possess anxiolytic activity supporting its traditional claim about anxiolytic activity reported in 19th edition of Pharmacology and Pharmacotherapeutics by Sathodkar, Bhandarkar and Rege.

### 4.6. Antistress Activity

Drug and food of natural origin play a significant role in public healthcare systems and are being investigated as remedies for a number of stress-related disorders [103]. The whole plant extract of* F. vulgare* exhibited notable antistress effect against stress induced by forceful swimming of test animals. The key parameters, that is, urinary levels of vanillyl mandelic acid (VMA) and ascorbic acid in rats were used to evaluate antistress activity. The plant extract (50, 100 and 200 mg/kg body weight) showed a significant improvement in urinary levels of VMA (*P* < 0.001), and ascorbic acid excretion levels (*P* < 0.001), in test animals when compared to the normal basal levels in control group of animals. Thus, the extract of entire plant of* F. vulgare *acts as an antistress agent [13].

### 4.7. Memory-Enhancing Property

There are a number of plants, whose consumption is believed to enhance memory and intelligence. These were usually given to children as part of their food.* F. vulgar*is an ayurvedic rasayana (mixture) possessing multiple neuropharmacological activities. The antidepressant activity of fennel has been well documented in ethnomedicine. The whole plants extract (50, 100 and 200 mg/kg) of* F. vulgare* exhibited memory-enhancing effect against scopolamine-induced amnesic rats. This experiment was evaluated by conditioned avoidance response (CAR) technique. The CAR of rats administered with the extract increased gradually to 95% over 7 to 12 days. The acquisition (time to achieve 95% CAR) for rats administered with the extract was dose- and time-dependent compared to control group, which took 12 days for acquisition. The percent avoidance was always higher in the extract-treated groups as compared to control group. Animals receiving 200 mg/kg body weight of the extract took ten days, while groups treated with 100 and 50 mg/kg doses of the extract required eleven and twelve days, respectively, to reach the point of acquisition. Administration of scopolamine produced amnesia as seen from reduction in the observed CAR. Amnesia was greater in the control group than in extract-treated groups. However, continued treatment with* F. vulgare *produced better retention and recovery in a dose-dependent manner than the vehicle-treated animals. Recovery from scopolamine-induced amnesia in the extract-treated groups took 3–5 days when compared to normal (control) group which took over 6 days. This overall progress suggests that* F. vulgare* extract possesses memory-enhancing property [13].

### 4.8. Nootropic Activity

Alzheimer's disease is a neurodegenerative disorder associated with a decline in cognitive abilities. Dementia is one of the age-related mental problems and a characteristic symptom of Alzheimer's disease. There is some evidence in favor of use of* F. vulgare* for the treatment of cognitive disorders like dementia and Alzheimer's disease. Methanol extract of the whole plant of* F. vulgare* administered for eight successive days ameliorated the amnesic effect of scopolamine and aging-induced memory deficits in mice. This extract increased step-down latency and acetylcholinesterase inhibition in mice significantly. Thus,* F. vulgare* may be employed in treatment of cognitive disorders such as dementia and Alzheimer's disease as a nootropic and anticholinesterase agent [80].

### 4.9. Antihirsutism Activity

Idiopathic hirsutism is defined as the occurrence of excessive male pattern hair growth in women who have a normal ovulatory menstrual cycle and normal levels of serum androgens. It may be a disorder of peripheral androgen metabolism. Traditionally,* Foeniculum vulgare* has been used as an estrogenic agent. It has been reputed to increase milk secretion, promote menstruation, facilitate birth, and increase libido. On considering above aspect, Javidnia and his research team evaluated the antihirsutism activity of ethanolic extract of* F. vulgare *seed against idiopathic hirsutism by preparing cream containing 1 and 2% of fennel extract. The efficacy of treatment with the cream containing 2% fennel is better than the cream containing 1% fennel and these two were more potent than placebo (control group). The mean values of hair diameter reduction were 7.8%, 18.3%, and −0.5% for patients receiving the creams containing 1%, 2%, and 0% (placebo), respectively [78].

### 4.10. Estrogenic Properties

Since the discovery of the estrus inducing effects of some plant products in 1926, considerable effort has been devoted towards the characterization of phytoestrogens, including flavonoids, isoflavonoids, chalcones, coumestans, stilbenes, lignans, saponins, and essential oils [16].* F. vulgare* has estrogen-like activity. In male rats, total concentration of protein was found to be significantly decreased in testes and in vasa deferentia whereas increased in seminal vesicles and in prostate gland. On the other hand, simultaneous decrease in the activities of acid and alkaline phosphatase in all these regions (except that alkaline phosphatase was unchanged in vasa), due to the oral administration of acetone extract of* F. vulgare* fruit, was observed. In female rats oral administration of the extract for 10 days led to vaginal cornification and oestrus cycle [8]. Total concentration of nucleic acids and protein as well as the organ weights increased in both the tissues, namely, mammary glands and oviducts, due to the oral administration of acetone extract (50, 150, and 250 *μ*g/100 g body wt) of* F. vulgare *seeds [104]. Fennel oil was reported to exhibit estrogenic activity, promote menstruation and alleviate the symptoms of female climacteric, and increase libido [71]. Administration of fennel oil (25 and 50 *μ*g/mL final concentration in the organ bath) failed to exhibit any remarkable effect in uterine contraction. While 10, 20 and 40 *μ*g/ml concentration of fennel oil revealed significant inhibitory effect against prostaglandin E2. Fennel oil significantly reduces the frequency of uterine contraction induced by prostaglandin E2. Thus, the extracts of* F. vulgare *have strong estrogenic activity [76].

### 4.11. Galactogenic Activity

*Foeniculum vulgare *has been used for millennia to increase milk secretion [105]. Thus,* F. vulgare* belongs to galactagogue substance. Structural similarity of its main constituent, anethole, to dopamine seems to be responsible for galactogenic activity. Dopamine acts to inhibit the secretion of the milk-producing hormone, prolactin. Anethole might influence milk secretion by competing with dopamine at the appropriate receptor sites, thereby inhibiting the antisecretory action of dopamine on prolactin [71]. It was reported that anol (demethylated anethole) causes growth of the lobule-alveolar system in the mammary glands of immature female rabbits and induces menstruation in mice and other experimental animals. Anol also gave positive results in the Jadassohn nipple test, a test which involves the measurement of changes induced in the nipples of guinea pigs subjected to the cutaneous application of sex hormones. However, further research suggests that the actual pharmacologically active agents responsible for galactogenic activity are polymers of anethole, such as dianethole and photoanethole, rather than anol or anethole itself [20, 76].

### 4.12. Expectorant Activity

*F. vulgare *seeds stimulate the ciliary motility of the respiratory apparatus and enhance the external transport of extraneous corpuscles. This action suggests a use for fennel in treating bronchial and bronchopulmonary afflictions and in particularly polluted environments [106]. The volatile oil of* F. vulgare *stimulates the contraction of the smooth muscles of the trachea, an action that could facilitate the expectoration of mucus, bacteria, and other corpuscles extraneous to the respiratory tracts [74].

### 4.13. Anticolitic Activity

Essential oil of fennel regulates the motility of smooth muscles of the intestine, while, at the same time, reducing intestinal gas. Alone, or combined with other plant medicinals,* Foeniculum vulgare *is indicated in the treatment of spastic gastrointestinal disturbances, in some forms of chronic colitis (which resist other treatments), in dyspepsias from gastrointestinal atony, in dyspepsias with the sensation of heaviness in the stomach, and so forth.The addition of fennel to preparations containing anthraquinonic components reduces the occurrence of abdominal pain often associated with this type of laxative [73].

### 4.14. Antinociceptive Activity

Antinociceptive means any substance that inhibits nociception which is a physiological process underlying the sensation of pain. Briefly, it reduces the sensitivity to painful stimuli. The various extracts of aerial part of* F. vulgare, *namely, hexane, methylene chloride, ethyl acetate, and methanolic extract showed remarkable antinociceptive activity against acetic acid induced writhing in mice [68]. The methanolic extract of the aerial parts of* F. vulgare *exhibited the highest antinociceptive activity at a dose level of 2000 mg/kg, while the activity exhibited by the ethyl acetate extract was at dose level 800 mg/kg. On the other hand,* n*-hexane extract (700 mg/kg) and methylene chloride extract (500 mg/kg) exhibited similar antinociceptive activities, being less than peripheral antinociceptive reference drug (acetyl salicylic acid) [68].

### 4.15. Diuretic Activity

A diuretic is any substance that promotes the production of urine. Briefly, it is an agent that promotes diuresis. Diuretics work by promoting the expulsion of urine (measured as the urine volume [UV] excreted) and urinary sodium (UNa) from the body and this helps reduce the volume of blood circulating through the cardiovascular system. Caceres et al. [107] performed a study in conscious animals and administered a powdered extract of the whole plant (*F. vulgare*) which had no effect on UV or UNa. The ethanolic extract of* F. vulgare* fruit revealed excellent diuretic activity and proves the earlier folk claim of* F. vulgare*, which was reported in the United State of America (Table 5). The fruit extract showed, statistically, a highly significant diuretic effect.* F. vulgare* induced diuresis (500 mg/kg dose) was comparable to that of reference diuretic agent urea (960 mg/kg dose) in mice with a urine output that was almost double that of the control group. The diuresis was not associated with changes in sodium and/or potassium excretion [108]. In another part of the study, the authors showed that* Foeniculum vulgare *had little effect on the noradrenalin contractile responses of aortic rings, thus suggesting that it worked mainly as a diuretic and natriuretic with little effect on arterial vascular tone [77].

### 4.16. Cardiovascular Activity

An aqueous extract of* F. vulgare* leaves possesses potential cardiovascular action. This effect was investigated using pentobarbital-anaesthetised male albino Sprague-Dawley rats [75]. An intravenous administration of the lyophilized boiled water extract of leaves produced a significant dose-related reduction in arterial blood pressure, without affecting the heart rate or respiratory rate. On the other hand the nonboiled aqueous extract showed very little hypotensive activity. The hypotensive effect of the boiling water extract appeared not to be mediated via adrenergic, muscarinic, ganglionic, or serotonergic receptors; however, histamine antagonists inhibited the hypotensive effect in a dose-related manner [75].

### 4.17. Oculohypotensive Activity

The aqueous seed extract of* F. vulgare* demonstrated significant oculohypotensive activity using water loading and steroid induced glaucoma model. This extract exhibited 17.49, 21.16, and 22.03% reduction of intraocular pressure in normotensive rabbits at 0.3%, 0.6%, and 1.2% (w/v) concentrations, respectively. A maximum mean difference of 31.20% was observed between vehicles treated and extracts treated eyes in water loading experimental animal model while a maximum mean intraocular pressure lowering of 31.29% was observed in steroid induced model of glaucoma. Thus, the aqueous extract of* F. vulgare* revealed oculohypotensive activity, which was found to be as good as that of reference standard antiglaucoma drugs called timolol [84].

### 4.18. Antithrombotic Activity

Tognolini et al. [55] provided evidence of potent inhibitory activity of essential oil of* F. vulgare *against platelet aggregation induced by ADP, arachidonic acid, and collagen in guinea pig plasma. Similar findings were reported by Yoshioka and Tamada [109] for aggregation of rabbit platelets by an aromatic factor of fennel oil. The essential oil and anethole (a constituent of oil) of* F. vulgare,* tested* in vitro* in rat aorta with or without endothelium, displayed comparable NO-independent vasorelaxant activity at antiplatelet concentrations. It supports the safety of* F. vulgare*, that is, free from cytotoxic effects. Anethole and* F. vulgare *did not cause cytotoxicity when incubated for 30 min upto 300 *μ*g/mL in platelet viability test. This concentration was largely compatible with those adopted in the functional* in vitro *tests.* In vivo*, both* F. vulgare *essential oil and anethole orally administered in a subacute treatment to mice (30 mg/kg/day for 5 days) showed significant antithrombotic activity preventing the paralysis induced by collagen-epinephrine intravenous injection (70% and 83% protection, resp.). Thus, essential oil and its main component anethole of* F. vulgare* showed a safe antithrombotic activity in guinea pig plasma that seems due to their broad spectrum antiplatelet activity, clot destabilizing effect, and vasorelaxant action [55].

### 4.19. Antimutagenic Effect

Essential oil of* F. vulgare *revealed noteworthy protective effects against genotoxicity in mice induced by cyclophosphamide. Genotoxicity and cytotoxicity were assessed by using mice bone marrow chromosomal aberration, micronucleus, and sperm abnormality assays, respectively. Oral administration of essential oil (1 and 2 mL/kg) significantly inhibited the frequencies of aberrant metaphases, chromosomal aberrations, micronuclei formation, and cytotoxicity in mouse bone marrow cells induced by cyclophosphamide and also produced a significant reduction of abnormal sperm and antagonized the reduction of cyclophosphamide induced superoxide dismutase, catalase, and glutathione activities and inhibited increased malondialdehyde content in the liver. Additionally,* F. vulgare* inhibits the oxidative stress induced by cyclophosphamide [90].

### 4.20. Gastrointestinal Effect

The aqueous extract of* F. vulgare *showed remarkable antiulcerogenic effect against ethanol-induced gastric lesions in rats. It was found that pretreatment with aqueous extract significantly reduced ethanol-induced gastric damage. This effect of aqueous extract was highest and statistically significant in 300 mg/kg group compared with the control (*P* < 0.001) group of animal. Additionally, aqueous extract of* F. vulgare *significantly reduced the whole blood malondialdehyde levels, while significantly increased nitrite, nitrate, ascorbic acid, retinol, and beta-carotene levels. Thus, aqueous extract of* F. vulgare* fruit had clearly a protective effect against ethanol-induced gastric mucosal lesion in rats [81].

### 4.21. Chemomodulatory Action

The chemopreventive effect of different doses of test diet of* Foeniculum vulgare* seeds was examined against 7,12-dimethylbenz(a)anthracene- (DMBA-) induced skin papillomagenesis and benzo(a)pyrene- [B(a)P-] induced forestomach papillomagenesis, at the peri-initiational level in Swiss albino mice. Fennel seeds exhibit a significant reduction in the skin and the fore-stomach tumor incidence and tumor multiplicity as compared to the control group. Further, biochemical assays showed a significant increase in the content/activities of phase I enzymes especially in the case of 6% test diet. A concomitant increase in the activities of the phase II enzymes was observed with all the doses of test diet under study. A significant enhancement in the activities of antioxidant enzymes was observed especially at 4% and 6% test diets of fennel. These findings were indicative of chemopreventive potential of fennel against carcinogenesis. This is the first report showing chemopreventive potential of seeds of fennel against carcinogenesis [85].

### 4.22. Cytoprotection and Antitumor Activity

Anethole is the principal active component of fennel seeds which has exhibited anticancer activity. Al-Harbi et al. studied the antitumor activity of anethole against Ehrlich ascites carcinoma induced in a tumor model in Swiss albino mice. The study revealed that anethole increased survival time, reduced tumor weight, and reduced the volume and body weight of the Ehrlich ascites tumour-bearing mice. It also produced a significant cytotoxic effect in the Ehrlich ascites tumour cells in the paw, reduced the levels of nucleic acids and malondialdehyde, and increased glutathione concentrations [110].* In vitro *cytoprotection activity of methanolic extract of* Foeniculum vulgare *was evaluated against normal human blood lymphocytes by micronucleus assay and antitumor activity against B16F10 melanoma cell line by Trypan blue exclusion assay for cell viability. Lymphocyte culture treated with 70% methanolic extract of* Foeniculum vulgare *showed very less percentage of micronucleus, that is, 0.006% as compared to standard drug doxorubicin which showed 0.018% micronucleus. On the other hand 70% methanolic extract of* Foeniculum vulgare *has potent antitumor activity at the concentration of 200 *μ*g/mL. The results suggest that the* Foeniculum vulgare *could be considered as a natural resource of antitumor agents as well as cytoprotective to normal cells [11].

### 4.23. Cytotoxicity

Kaileh and his coworker investigated the cytotoxic effect of organic extracts of 24 selected Palestinian medicinal plant species. The plant selection was based on existing ethnobotanic information and interviews with local healers. The extracts of the plants under investigation were tested for their potential antitumor (cytotoxic) effect on the murine fibrosarcoma L929sA cells and on the human breast cancer cells MDA-MB231 and MCF7. The extract from* F. vulgare *presented an IC (50) value at 24 h of 700 ± 28 and 500 ± 17 *μ*g/mL, on L929sA and MCF7 cells, respectively. The nuclear transcription factor NFkappaB or NF*κ*B regulates the expression of various genes. They further investigated the effect of nine promising plant extracts, withheld from the first cell viability screening on NF*κ*B activation. The dichloromethane and methanol (1 : 1) extract of aerial part of* F. vulgare* revealed immunomodulatory NF*κ*B activities [82]. Also, Berrington and Lall investigated the* in vitro *cytotoxicity of acetone extracts of* F. vulgare *and other eight medicinal plants against a noncancerous African green monkey kidney (Vero) cell line and an adenocarcinoma cervical cancer (HeLa) cell line [87].

### 4.24. Antipyretic Activity

*F. vulgare* extract showed antipyretic activity against hyperpyrexia in mice. It was induced by S.C. administration of 2 mL/100 g of a 20% aqueous suspension of brewer's yeast. As an antipyretic agent, ethanolic extract of* F. vulgare *fruit showed a moderate antipyretic activity that was statistically significant after 30 and 90 min (*P* < 0.01) [108].

### 4.25. Hypolipidemic Activity

The aqueous extract of* F. vulgare *revealed notable hypolipidemic and antiatherogenic activity against Triton WR-1339 induced hyperlipidemia in mice. Aqueous extract causes significant reduction of plasma lipid levels, that is, cholesterol, triglycerides, LDL-cholesterol, and apolipoprotein-B decreased by 40%, 23%, 61%, and 61%, respectively, and increase in HDL-cholesterol and apolipoprotein A1 by 85% and 58%, respectively [88].

### 4.26. Hypoglycemic Activity

The essential oil of* F. vulgare* exhibits potential hypoglycemic and antioxidant activity against streptozotocin induced diabetes in rats. Essential oil (30 mg/kg body weight) of* F. vulgare* works in the correction of hyperglycemia from 162.5 ± 3.19 mg/dL to 81.97 ± 1.97 mg/dL with *P* < 0.05 and the activity of serum glutathione peroxidase from 59.72 ± 2.78 U/g Hb to 99.60 ± 6.38 U/g Hb with *P* < 0.05. Also, essential oil of fennel improves the pathological changes noticed in their kidney and pancreas as compared with the control group of animal. This can prove its effect as antidiabetic in folk Medicine. This makes the possibility of its inclusion in antidiabetic drug industry [12].

### 4.27. Antispasmodic Activity

The antispasmodic activity of 2.5 and 10.0 mL/L of alcoholic extract of* Foeniculum vulgare* along with other Germanic medicinal plants, namely,* Melissa officinalis*,* Rosmarinus officinalis*,* Mentha piperita*,* Matricaria chamomilla*,* Carum carvi,* and* Citrus aurantium* were tested employing the guinea pig ileum and using acetylcholine and histamine as spasmogens. An alcoholic extract of the fruits of* Foeniculum vulgare* possesses antispasmodic activity, which inhibits the acetylcholine and histamine-induced guinea pig ileal contractions* in vitro*. An essential oil which is obtained from the fruits of* Foeniculum vulgare*, 25 *μ*g/mL and 10 *μ*g/mL, respectively, inhibited oxytocin and prostaglandin [72].

### 4.28. Apoptotic Activity

The apoptotic activities of ethanol extracts from fruits of seven species of Apiaceae family, namely,* Eryngium planum*,* Archangelica officinalis*,* Pastinaca sativa*,* Heracleum sibiricum*,* Carum carvi*,* Foeniculum vulgare,* and* Levisticum officinale* against ML-1—human acute myeloblastic leukaemia, J-45.01—human acute T cell leukaemia, EOL—human eosinophilic leukaemia, HL-60—human Caucasian promyelocytic leukaemia, 1301—human T cell leukaemia lymphoblast, C-8166—human T cell leukaemia, U-266B1—human myeloma, WICL—human Caucasian normal B cell, and H-9—human T cell were investigated with the help of Trypan blue assay and Annexin V fluos assay [86]. The ethanol extract from fruit* F. vulgare* showed the highest mortality in Trypan blue test for J45 cell line—4% of viable cells and for C8166 cell line—100% of mortality. However the cells of other lines showed the highest viability: HL60—60%, EOL—48%, and ML—1–42%. The normal cell line H9 and WICL showed 35% and 25% of viable cells, respectively. C8166 cell line and J-45 cell line showed the highest level of the apoptotic cells detected by Annexin V method—100% and 93%, respectively. However the cells of two lines HL60 and EOL-1 showed the lower levels of apoptotic cells—52% and 60%, respectively. High percentage of apoptotic cells was observed in H9 and WICL—76% and 93%, respectively [86].

### 4.29. Human Liver Cytochrome P450 3A4 Inhibitory Activity

Thirteen compounds isolated from the methanolic extract of fennel have been found to possess human liver cytochrome P450 3A4 inhibitory activity. Among these compounds 5-methoxypsoralen (5-MoP) showed the strongest inhibition with an IC50 value of 18.3 *μ*m and with a mixed type of inhibition [83].

### 4.30. Antiaging Effects

Rasul and his coworker developed a base and formulation containing 4% concentrated seed extract of* F. vulgare*. This formulation shows notable antiaging effect with supporting experimental data related to skin moisture and transepidermal water loss (TEWL). The base was insignificant, while the formulation showed significant effects on skin moisture and TEWL. The texture parameter energy showed a significant increase proving that the formulation possesses potential antiaging effects [50].

### 4.31. Bronchodilatory Effect

Ethanol extract and essential oil from* F. vulgare *exhibited bronchodilatory activity on contracted tracheal chains of guinea pig. The potassium channel opening effect of fennel may contribute on its relaxant effect on guinea pig tracheal chains [111]. Moreover, anethole bears a striking resemblance to the catecholamines epinephrine, norepinephrine, and dopamine. This structural similarity appears to be responsible for the various sympathomimetic activities of* F. vulgare* such as bronchodilatory effect [71].

### 4.32. Antioxidant Activities

Naturally occurring antioxidants can be used to protect human beings from oxidative stress damage [112]. Fennel was known as excellent source of natural antioxidants and contributed to the daily antioxidant diet [113]. Wild fennel was found to exhibit a free radical scavenging activity with higher content phenolic and flavonoid than medicinal and edible fennel, and the aerial parts of the Italian fennel populations showed the highest DPPH scavenging activity [65]. Phenolic compounds of fennel, including caffeoylquinic acid, rosmarinic acid, eriodictyol-7-orutinoside, quercetin-3-*O*-galactoside, and kaempferol-3-*O*-glucoside, showed antioxidant activities [67]. The volatile oil showed strong antioxidant activity in comparison with butyrated hydroxyanisole and butylated hydroxytoluene. Ethanol and water extracts of fennel showed less antioxidant activity compared with essential oil [114].

## 5. Environmental Application

*Foeniculum vulgare,* that is, fennel, not only exhibited pharmacological activities but also revealed a few environmental activities. These activities play a key role in the management of nematode, insect, mosquitoes, and some harmful larvae of malaria producing vector. Thus, the extracts of* F. vulgare *and isolated biologically active compounds have been evaluated for their insecticidal, repellent, acaricidal, larvicidal, and nematicidal activity [115–119]. A brief review on the different type of ecofriendly environmental activities as reported on this plant is summarized below.

### 5.1. Insecticidal Activities

The fruit derived phytoconstituents of* F. vulgare* exhibited prominent insecticidal activities against* Sitophilus oryzae*,* Callosobruchus chinensis,* and* Lasioderma serricorne*. This activity was examined using direct contact application and fumigation methods. The biologically active constituents, that is, phenylpropenes (E)-anethole and estragole, and the monoterpene (+)-fenchone were characterized from* Foeniculum* fruit. By using a filter paper diffusion test, estragole (0.168 mg cm^−2^) caused 91% mortality to* S. oryzae* within 1 day after treatment whereas (+)-fenchone and (E)-anethole gave over 90% mortality at 2 and 4 day after treatment, respectively.

After 2 days of treatment, all test compounds (0.021 mg cm^−2^ concentration) revealed potent insecticidal activity against* C. chinensis*. Whereas after 1 day of treatment, (E)-anethole (0.105 mg cm^−2^) gave 100% mortality of* L. serricorne* whereas 90 and 60% mortality at 4 day after treatment was achieved with estragole and (+)-fenchone, respectively. In a fumigation test, the compounds were much more effective against adults of* S. oryzae*,* C. chinensis,* and* L. serricorne* in closed cups than in open ones, indicating that the insecticidal activity of test compounds was largely attributable to fumigant action. As naturally occurring insect-control agents, the* F. vulgare* fruit-derived materials described could be useful for managing field populations of* S. oryzae*,* C. chinensis,* and* L. serricorne *[116].

### 5.2. Acaricidal Activity

Fennel oil shows significant acaricidal activity against* Dermatophagoides farinae *and* Dermatophagoides pteronyssinus*. (+)-fenchone and p-anisaldehyde are major constituents of fruit oil of* F. vulgare*.* P-*anisaldehyde was the most toxic compound against* D. farinae *and is much more effective compared with benzyl benzoate, thymol, and estragol [118].

### 5.3. Repellent Activity

The methanolic extract of fruits of* F. vulgare* was spectroscopically characterized for the presence of biologically active constituents called (+)-fenchone and (E)-9-octadecenoic acid. The repellent activity of these constituents was tested against hungry* Aedes aegypti* females with the help of skin and patch tests and compared with that of the commercial repellent agent called N,N-diethyl-m-toluamide (DEET) and (Z)-9-octadecenoic acid. In a skin test with female mosquitoes (+)-fenchone and (Z)-9-octadecenoic acid (0.4 mg/cm^2^) exhibited moderate repellent activity at 30 min after treatment, whereas DEET provided >1 h of protection against adult mosquitoes at (0.2 mg/cm^2^). Thus, (+)-Fenchone and (E)-9-octadecenoic acid are potential mosquito repellent agents or lead compounds [117].

### 5.4. Larvicidal Activity

Plant extracts and oils may act as alternatives to conventional pesticides for malaria vector control. By considering this aspect, Sedaghat et al. [119] investigated the larvicidal activity of essential oils of three plants of* Apiaceae* family against malaria vector called* Anopheles stephensi*. The larvicidal activity was evaluated against laboratory-reared larvae by standard method of WHO. The* F. vulgare* oil was the most effective against* A. stephensi* with LC(50) and LC(90) values of 20.10 and 44.51 ppm, respectively [119]. Additionally, the essential oil extracts from leaves, flowers, and roots of* F. vulgare* exhibit noticeable larvicidal activity against fourth-instar larvae of the mosquito* Culex pipiens molestus*. Terpineol and 1,8-cineole content of* F. vulgare *are the most effective phytoconstituent against* Culex pipiens molestus *bites offering complete protection for 1.6 and 2 h, respectively [120]. Recently, Zoubiri et al. [57] reported the larvicidal activity of essential oil of fennel seed against* Culex pipiens *mosquito. Thus,* F. vulgare* can serve as a natural larvicidal agent.

### 5.5. Nematicidal Activity

Oka et al. investigated the* in vitro* nematicidal activity of essential oils extracted from 27 spices and aromatic plants in pot experiments. Twelve of the twenty-seven essential oils immobilized more than 80% of juveniles of the root-knot nematode* Meloidogyne javanica *at a concentration of 1000 *μ*L/liter. At this concentration, most of these oils also inhibited nematode hatching. Essential oils of* Carum carvi*,* Foeniculum vulgare, Mentha rotundifolia*, and* Mentha spicata *showed the highest nematicidal activity among the* in vitro* tested oils. In 3-liter pot experiments, nematicidal activity of the essential oils and their components was confirmed at 200 and 150 mg/kg, respectively. The results suggest that the essential oils and their main components may serve as nematicides [115].

## 6. Toxicity

The long history of ethnomedicinal application, with no reports of any serious side effects, suggests that* F. vulgare *could be considered as safe. In most toxicity experiments carried out on* F. vulgare, *no sign of toxicity was observed. Shah and his coworker in 1991 investigated the detailed toxicity account of ethanolic extract of fennel fruit in experimental mice with respect to acute and 90 days longer term toxicity [121]. In experimentation, Shah and his coworker observed the general symptoms of toxicity and mortality for only 24 h in acute toxicity. Whereas, in another part of toxicity they studied the effect of fennel extract on mice with 90 days long term treatment. Acute toxicity of ethanolic extract of* F. vulgare* was assessed in 35 mice by using three concentrations, namely, 0.5, 1, and 3 g/kg body weight. In this investigation,* F. vulgare *exhibited no signs of toxicity and no mortality was observed upto the dose level 3 g/kg body weight. In case of longer term toxicity, ethanolic extract of* F. vulgare* (100 mg/kg body weight/day) was given in drinking water of animals (30 male and 30 female mice). All external morphological, haematological, and spermatogenic changes, in addition to body and vital organ weights, were recorded. The extract caused no significant chronic mortality as compared to controls during this investigation. The treated male mice gained significant weight during chronic treatment while a loss or no significant change in weight was noticed in the female mice treated with the same extract. The extracts did not show spermatotoxic effects. Thus, Shah and his coworker concluded that fennel extract is safe based on both acute and/or long term toxicity studies [121]. Additionally, the plant extract in doses of 0.5, 1, and 3 g/kg (orally) did not cause any deaths. These doses do not show any type of toxicity against several parameters tested, namely, locomotor activity, bizarre reactions, sensitivity to sound, social interaction, tail posture, aggressive behaviour, ataxia, paralysis, convulsions, tremors, prostration, exophthalmos, pupil size, defecation, salivation, urination, pattern of respiration, nasal discharge, cyanosis, and piloerection. Exceptionally, only the 3 g/kg dose showed signs of reduced locomotor activity and piloerection. Otherwise, all other parameters were negative [108]. In another experiment of acute toxicity, different solvent extracts, namely,* n*-hexane, methylene chloride, ethyl acetate, and methanol extracts of* F. vulgare* upto 5.5 g/kg concentration, did not revealed any kind of toxicity in mice, LD50 being: 6.75, 11.0, 6.92, and 15 g/kg for* n*-hexane, methylene chloride, ethyl acetate, and methanol extracts, respectively [68]. The plant extract of* F. vulgare* was administered orally at a dose of 100, 200, 400, 600, 800, 1000, and 2000 mg/kg of body weight of mice. Each group of animals was under visual observation for 10 days for the external behavior of neurological toxicity created by plant extract. Even the mice receiving highest dose of* F. vulgare* extract did not show any mortality or toxicity demonstrating the safety profile of the plant extract [89].

The acute oral 50% LD50 for anethole in rats was found to be 2090 mg/kg. Repeated doses of one-third the LD50 of anethole (695 mg/kg) given to rat caused mild liver lesions. It would therefore appear that in normal therapeutic dosages anethole would have minimal hepatotoxicity. When anethole was fed to rats daily for one year as 0.25% of the diet, no hepatic damage was seen [122]. The acute oral LD50 of essential oil in rats is 1326 mg/kg [76]. The use of* F. vulgare* essential oil as a remedy for control of primary dysmenorrhea increases concern about its potential teratogenicity due to its estrogen like activity. Evaluation of teratogenicity of essential oil using limb bud mesenchymal cells showed that the essential oil may have toxic effect on fetal cells, but there was no evidence of teratogenicity upto concentration of 9.3 mg/mL of culture medium [123]. The overall toxicity studies carried out on* F. vulgare *accounts for its safety at the recommended therapeutic doses.

## 7. Conclusions

The available scientific research on* Foeniculum vulgare *has shown that it is an important medicinal plant used in a wide range of ethnomedical treatments, especially for abdominal pains, antiemetic, aperitif, arthritis, cancer, colic in children, conjunctivitis, constipation, depurative, diarrhea, dieresis, emmenagogue, fever, flatulence, gastralgia, gastritis, insomnia, irritable colon, kidney ailments, as a laxative, leucorrhoea, liver pain, mouth ulcer, and stomachache. This plant has been in use for a long period of time without any documented serious adverse effects. Studies carried out in the past and present indicate that fennel possesses diverse health benefits and are an important constituent of food. Studies have shown that various extracts of fennel possess a range of pharmacological actions, such as antiaging, antiallergic, anticolitic, antihirsutism, anti-inflammatory, antimicrobial and antiviral, antimutagenic, antinociceptive, antipyretic, antispasmodic, antistress, antithrombotic, anxiolytic, apoptotic, cardiovascular, chemomodulatory action, cytoprotection and antitumor, cytotoxicity, diuretic, estrogenic properties, expectorant, galactogenic, gastrointestinal effect, hepatoprotective, human liver cytochrome P450 3A4 inhibitory, hypoglycemic, hypolipidemic, memory-enhancing property, nootropic, and oculohypotensive activity supporting its traditional use. However, the most prominent and the well studied effects are the antimicrobial and antioxidant effects of essential oil of fennel in different experimental models. The observed health benefits may be credited to the presence of the various phytochemicals like volatile compounds, flavonoids, phenolic compounds, fatty acids, and amino acids.

Fennel also contains mineral and trace elements like aluminum, barium, calcium, cadmium, cobalt, chromium, copper, iron, magnesium, manganese, nickel, lead, strontium, and zinc [124]; fat soluble vitamins such as vitamins A, E, and K; water soluble vitamins like ascorbic acid, thiamine, riboflavin, niacin, and pyridoxine; essential amino acids like leucine, isoleucine, phenylalanine, and tryptophane may contribute to the myriad health beneficial effects at least in part.

Most of the pharmacological studies were conducted using uncharacterized crude extracts of fennel. It is difficult to reproduce the results of these studies and pinpoint the bioactive compounds. Hence, there is a need for chemical standardization and bioactivity-guided identification of bioactive compounds. Among several classes of chemical constituents identified in fennel, volatile components of fennel essential oil and phenolic compounds are assumed to be the main bioactive compounds responsible for the majority of its pharmacological effects. However, the vast traditional use and proven pharmacological activities of fennel indicate that an immense scope still exists for its chemical exploration. Future studies should be focused on validating the mechanism of action responsible for the various beneficial effects and also on understanding which plant based compounds are responsible for the reported effects. The required information when available will enhance our knowledge and appreciation for the use of fennel in our daily diet. Also, the outcome of such chemical studies may further expand its existing therapeutic potential.

Thus, there are many areas of research related to this plant that need to be further explored to fully recognize its beneficial effects for society. Factors such as geographical and seasonal variation play an important role in the authentication of the chemical constituents responsible for the activity which also can be an area of interest. Thus, it is incumbent on researchers to fill the huge gap of insufficient knowledge and create awareness among pharmacologists as well as investigators towards providing better medicinal value derived from this plant. This can be fulfilled only by generating interest among the research community through writing of critical appraisals (paper) and extending the interdisciplinary research area to focused studies on* Foeniculum vulgare*.

## Figures and Tables

**Table 1:** Vernacular names of *Foeniculum vulgare*.

Region/language/system of medicine	Local name
Alto, Bolivia	Hinojo
Arabic	Bisbas, razianaj
Aymara, Kechua	Inuju
Balikesir, Turkey	Arapsaci, rezene, malatura, hullebe
Basque	Mieloi
Bengali (Indian language)	Mauri, pānmourī
Bosnia	Komorač
Brazil	Endro, erva-doce, funcho
Catalan	Fenoll, fonoll
Central Serbia	Morac
Chinese	Hui xiang, xiao hui xiang
Czech	Fenykl
Dalmatia (southern Croatia), Poland	Komorač, koromač, kumurač, morač, moroč, morača, Koper wloski
Danish	Almindelig fennikel, fennikel
Denmark	Almindelig
Dutch	Venkel
English	Bitter fennel, common fennel, sweet fennel, wild fennel
France	Fenouille
French	Fenouil
Germany	Fenchel, fenchle, bitterfenchel, wilder fenchel, dunkler fenchel,
Guerrero, Mexico	Hinojo
Gujarati (Indian language)	Hariyal, variyali
Haryana, India	Saunf
Hindi (Indian language)	Badi, badishep, bari saunf, badi saunf, saunp, saunf, sonp, sont
Italy	Finucchio, finucchiello, finochietto, finocchiella, fenùcciu, fenucéttu-sarvègu
Jammu and Kashmir, India	Saunf
Japanese	Fenneru, uikyou, uikyou, shouikya
Java, Indonesia	Adas
Jordan	Shomar
Kallawaya	Jinuchchu
Kannada	Badi sopu, badisepu, sabbasige, dodda sopu, dodda jirige
Korea	Sohoehyang
Laotian	Phaksi
Latin	Foeniculum, maratrum
Loja, Ecuador	Hinojo
Majorcan area	Fonoll
Middle Navarra	Hinojo, cenojo
Marathi (Indian language)	Badishep, ba*ḍ*īśep, shoap
Nepalese	Madesi sauf
North Iran	Badian
North Portugal	Funcho
Norway	Fenikkel
Norwegian	Fennikel
Pakistan	Sonef, saunf
Peninsula, Spain	Hinojo
Persian	Razianeh
Polish	Fenkuł, koper włoski
Portuguese	Funcho
Rajasthan, India	Sanuf
Sanskrit (Indian language)	Madhurika, shatapushpa
Slovenian	Sladki komarček
Somali Region, Ethiopia	Kamon
South Europe	Fennel
South Africa	Vinkel, fennel
Spanish	Hinojo, hinojo amargo, fenoll, fiollo, millua
Swedish	Fänkål
Tamil (Indian language)	Perun siragum, shombu, sohikire
Telugu (Indian language)	Peddajilakurra, sopu
Thai	Phak chi, phak chi duen ha, phak chi lom, thian klaep, yira
Uttarakhand, India	Badesoppu

**Table 2:** Nutrients found in dried fennel (USDA, USA).

Composition	Quantity (Per 100 g)
Proximates	
Moisture	90.21 g
Energy	31 kcal
Protein	1.24 g
Total lipid (fat)	0.2 g
Carbohydrate	7.3 g
Total dietary fiber	3.1 g
Sugars	3.93 g
Minerals	
Calcium, Ca	49 mg
Iron, Fe	0.73 mg
Magnesium, Mg	17 mg
Phosphorus, P	50 mg
Potassium, K	414 mg
Sodium, Na	52 mg
Zinc, Zn	0.2 mg
Vitamins	
Vitamin C	12 mg
Thiamin B-1	0.01 mg
Riboflavin B-2	0.032 mg
Niacin B-3	0.64 mg
Vitamin B-6	0.047 mg
Folate	27 *μ*g
Vitamin A	48 *μ*g
Vitamin E	0.58 mg
Vitamin K	62.8 *μ*g
Lipids	
Fatty acids, total saturated	0.09 g
Fatty acids, total monounsaturated	0.068 g
Fatty acids, total polyunsaturated	0.169 g
Essential amino acids	
Leucine	0.63 g
Isoleucine	0.73 g
Phenylalanine	0.45 g
Tryptophane	0.53 g
Nonessential amino acid	
Glycine	0.55 g
Proline	0.53 g

**Table 3:** Nutrient content of different parts of *Foeniculum vulgare*.

Composition	Contents			
	Leaves	Inflorescences	Stems	Shoots
Moisture^a^	76.36 ± 0.33	71.31 ± 4.01	77.46 ± 1.03	73.88 ± 0.83
Ash^a^	3.43 ± 0.04	3.23 ± 0.02	1.62 ± 0.12	2.39 ± 0.02
Fat^a^	0.61 ± 0.16	1.28 ± 0.28	0.45 ± 0.07	0.49 ± 0.05
Protein^a^	1.16 ± 0.03	1.37 ± 0.05	1.08 ± 0.00	1.33 ± 0.04
Carbohydrates^a^	18.44 ± 0.06	22.82 ± 3.06	19.39 ± 0.65	21.91 ± 0.55
Fructose^a^	0.49 ± 0.05	1.10 ± 0.04	1.49 ± 0.04	1.51 ± 0.06
Glucose^a^	0.76 ± 0.12	2.94 ± 0.11	3.43 ± 0.20	4.71 ± 0.15
Sucrose^a^	0.04 ± 0.00	0.03 ± 0.00	nd	0.35 ± 0.06
Reducing sugars^a^	0.72 ± 0.04	1.20 ± 0.19	1.49 ± 0.29	1.14 ± 0.10
*ω*3 fatty acid^b^	43.72 ± 0.36	17.69 ± 0.01	23.04 ± 1.30	36.96 ± 0.51
*ω*6 fatty acid^b^	23.25 ± 0.07	38.94 ± 0.23	38.22 ± 0.68	39.99 ± 0.68
*ω*6/*ω*3	0.53 ± 0.00	2.20 ± 0.01	1.66 ± 1.12	1.08 ± 0.03
C6:0^b^	0.02 ± 0.00	0.41 ± 0.02	0.19 ± 0.01	0.06 ± 0.00
C8:0^b^	0.08 ± 0.00	0.37 ± 0.01	0.48 ± 0.03	0.33 ± 0.00
C10:0^b^	0.04 ± 0.00	0.09 ± 0.00	0.13 ± 0.01	0.06 ± 0.00
C11:0^b^	0.25 ± 0.02	0.29 ± 0.01	0.04 ± 0.00	0.07 ± 0.00
C12:0^b^	0.31 ± 0.02	0.43 ± 0.06	0.11 ± 0.01	0.21 ± 0.02
C14:0^b^	1.43 ± 0.01	1.68 ± 0.10	0.49 ± 0.06	0.75 ± 0.03
C14:1^b^	0.61 ± 0.04	0.28 ± 0.02	0.37 ± 0.04	0.17 ± 0.03
C15:0^b^	0.17 ± 0.00	0.35 ± 0.03	0.41 ± 0.04	0.18 ± 0.00
C16:0^b^	20.15 ± 0.09	23.89 ± 0.07	25.43 ± 0.00	12.78 ± 0.09
C17:0^b^	0.74 ± 0.00	0.58 ± 0.02	0.61 ± 0.04	0.24 ± 0.02
C18:0^b^	1.61 ± 0.08	2.62 ± 0.04	1.99 ± 0.06	1.53 ± 0.08
C18:1n9c^b^	4.35 ± 0.37	5.05 ± 0.00	4.35 ± 0.52	2.55 ± 0.33
C18:2n6c^b^	23.25 ± 0.07	38.94 ± 0.23	38.22 ± 0.68	39.99 ± 0.68
C18:3n3^b^	43.55 ± 0.40	17.55 ± 0.0	22.86 ± 1.31	36.84 ± 0.52
C20:0^b^	0.56 ± 0.00	1.78 ± 0.06	0.84 ± 0.03	1.06 ± 0.09
C20:1c^b^	nd	0.26 ± 0.03	0.06 ± 0.00	nd
C20:2c^b^	0.08 ± 0.01	0.31 ± 0.01	0.14 ± 0.00	0.38 ± 0.07
C20:3n3 + C21:0^b^	0.16 ± 0.02	0.15 ± 0.01	0.19 ± 0.00	0.12 ± 0.01
C22:0^b^	0.77 ± 0.04	1.52 ± 0.04	1.20 ± 0.03	1.12 ± 0.02
C23:0^b^	0.82 ± 0.13	1.89 ± 0.11	0.68 ± 0.01	0.36 ± 0.15
C24:0^b^	1.03 ± 0.04	1.58 ± 0.02	1.21 ± 0.02	1.20 ± 0.08
Total SFA^b^	27.99 ± 0.02	37.47 ± 0.25	33.81 ± 0.06	19.95 ± 0.12
Total MUFA^b^	4.96 ± 0.40	5.59 ± 0.13	4.78 ± 0.57	2.72 ± 0.36
Total PUFA^b^	67.05 ± 0.42	56.94 ± 0.12	61.41 ± 0.62	77.33 ± 0.24
Energy^c^	83.90 ± 1.34	108.23 ± 10.37	85.91 ± 3.02	97.37 ± 2.44

^a^Nutrients composition (g/100 g), ^b^ *ω*3 and *ω*6 and fatty acid content (percent), and ^c^energetic value (Kcal/100 g) of the different parts of fennel. nd: not detected. Values are expressed as mean ± SD, *n* = 3 experiments in each group [14].

**Table 4:** Uses of *Foeniculum vulgare* as a food ingredient as reported in the literature.

Sr. number	Region/Nation	Local name	Part used and edible application.	References
1	Campania, Italy	Finucchio, finucchiello, finochietto	Stem is used as an aromatizer for pickled olives.	[125]

2	Campania, Italy	Finocchiella, fenùcciu	Seed is employed in preparation of salted meats.	[125]

3	Spain	Hinojo, Fenoll	Tender leaves and stems, raw as a snack, are used in salads or stewed.	[126]

4	Spain	Fiallo, millau	Aerial part or seeds used for seasoning olives, as preservative for dry figs, and for preparing herbal tea or liqueur.	[126]

5	Trás-os-Montes (Northeast Portuguese)	Fialho, fionho, erva-doce	Shoots, tender leaves, and stems used in snacks, salads, soups, stews, and spices. Flowering stems used in beverages, spirits, and spices. Stems used as brochettes and herbal teas. Seeds used as spices, flavour for cakes, biscuits, and sweets, and chestnuts.	[14]

6	Arrábida and Açor (Center Portuguese)	Funcho, erva-doce	Seeds used as flavour for cakes and pastries and for cooking chestnuts.	[14]

7	Alentejo and Algarve (South Portuguese)	Funcho, fialho, funcho-doce, funcho-amargo	Shoots, tender leaves, and stems are fried with eggs, used in omelettes, used in fish stuff, stewed with different kinds of beans and chickpeas, and used in fish and bread bouillons, soups, and sauces. Tender leafy stems are used in grilled fish and fish dishes in general. Seeds are used as spices, flavour for cakes, bread, and biscuits, and chestnuts. Whole plant used in olives brines, figs preserves, and for aromatizing brandy.	[14]

8	Jammu and Kashmir, India	Saunf	The fruits with other ingredients are given to the animal if it stops taking food during diarrhea.	[46]

9	Liguria, Italy	Fenucéttu-sarvègu	Aerial parts of plant mixed with shoots of *Clematis *and *Rubus *used as food integrator for sheep.	[45]

**Table 5:** Traditional and contemporary applications of *Foeniculum vulgare*.

Sr. number	Ailment/use	Part/preparation used	Locality	References
1	Mouth ulcer	Tender leaves, chewed and stuck on ulcer	Basilicata, Italy	[33]

2	Aperitif	Tender parts-raw or boiled	Rome, Italy	[32]

3	Gum disorder	Fruit and seed, used as a mouth wash for gum disorder	Central Serbia	[35]

4	Insomnia	Infusion of tea leaf	Brazil	[31]

5	Constipation	Seeds, decoction	South Europe	[127]
		Seeds mixed with sugar	Jammu and Kashmir, India	[36]

6	Cancer	Leaf and flower, aqueous infusion, drink	Loja, Ecuador	[128]

7	Conjunctivitis	Leaf and flower, aqueous infusion, drink	Loja, Ecuador	[128]

8	Gastritis	Leaf, flower, aqueous infusion, drink	Loja, Ecuador	[128]

9	Diuresis	Root and seed, decoction	Miami, Florida, USA	[42]

10	Abdominal pains	Each plant part, decoction	Rome, Italy	[30]
		Leaf and seeds, infusion	Northern Badia, Jordan,	[39]
		Leaves, paste	Manisa, Turkey	[43]

11	Cold	Fruits and floral tops, decoction	Rome, Italy	[30]

12	Refreshing	Roots/whole plant, decoction	Rome, Italy	[30]

13	Swollen stomach	Leaves, decoction with a little honey	Rome, Italy	[129]

14	Hair grow	Seed oil	Middle Navarra	[130]

15	Antiemetic	Fruit, simple powder	Northeastern Majorcan area	[47]

16	Antihypertensive and Anti-cholesterolemic	Leaf directly chewed	north-eastern Majorcan area	[47]

17	Depurative	Leaf and stem, comestible	Iberian Peninsula, Spain	[40]

18	Hypnotic	Seed, leaf, and stem, infusion and edible	North Iran	[24]

19	Diarrhoea	Seeds, roots, and fresh leaves	Northern Portugal	[28]
		Seeds grounded with Root tubers of *Hemidesmus indicus *and the paste taken with jaggery twice a day for three days	Bhandara, Maharashtra, India	[131]

20	Kidney ailments	Aerial part, infusion	Alto, Bolivia	[34]
		Seed, decoction	Gujranwala, Pakistan	[132]

21	Colic in children	Leaf and fruit, infusion	Brazil	[133]

22	Irritable colon	Leaf and seeds, infusion	Northern Badia, Jordan,	[39]

23	Gastralgia	Leaf, decoction	southern Spain	[29]

24	Purgative	Seed, infusion and edible	Gujranwala, Pakistan	[132]

25	Laxative	Seed, infusion and edible	Gujranwala, Pakistan	[132]

26	Liver pain	Seed	Pernambuco, Northeast Brazil	[133]

27	Mosquitocidal	Root boiled and drunk as tea	Somali Region, Ethiopia	[41]

28	Arthritis	Leaf, an infusion made from the leaves is drunk	South Africa	[37]

29	Fever	Leaf, an infusion made from the leaves is drunk	South Africa	[37]

30	Fat deduction	Green fruit is chewed to reduce fat	South Africa	[37]

31	Leucorrhoea	A mixture of its 100 g seed powder, 200 g seed powder of *Papaver somniferum*, 100 g fruit powder of *Coriander sativum,* and 200 g of sugar is prepared and 50 g of this mixture is taken by the tribal ladies early in the morning	Rajasthan, India	[26]

32	Problem of repeated abortions	Mixture of its 50 g seed powder, 50 g fruit powder of *Trapa natans,* and 50 g sugar is given daily to pregnant ladies	Rajasthan, India	[26]

33	Digestive system	Fruits, decoction	Basilicata, Italy	[33]
		Seed, decoction (drink one tea cup after food)	Balikesir, Turkey	[134]
		Whole plant	Western cape of South Africa	[135]
		Fruit, powder for digestive ailments	Middle, West, and South Bosnia	[136]
		Seeds, decoction	South Europe	[127]
		Seeds, roots, and fresh leaves	Northern Portugal.	[28]
		Seed, decoction	Southern Spain	[29]

34	Carminative	Tender parts, raw or boiled	Rome, Italy	[32]
		Whole plant	Western cape of South Africa	[135]
		Seeds, decoction	South Europe	[127]
		Seed, leaf, and stem, infusion and edible	North Iran	[24]
		Leaves and/or fruits	South Africa	[27]

35	Diuretic	Tender parts, raw or boiled	Rome, Italy	[32]
		Whole plant	Western cape of South Africa	[135]
		Seeds, decoction	South-Europe	[127]
		Seeds, roots, and fresh leaves	Northern Portugal.	[28]
		Leaf, an infusion made from the leaves is drunk	South Africa	[37]

36	Emmenagogue	Aerial part, raw with carrot	Rome, Italy	[32]
		Fruit, simple powder	North-eastern Majorcan area	[47]
		Seed	Haryana, India	[137]

37	Milk stimulant in pregnant women (Galactagogue)	Leaf, an infusion made from the leaves is drunk	South Africa	[37]
		Fruits, as condiment or chewed	Rome, Italy	[32]
		Fruit, simple powder	north-eastern Majorcan area	[47]
		Aerial part-infusion	Alto, Bolivia	[34]

38	Gingival wound	Fruit-paste	Uttarakhand, India	[138]
		Whole plant, decoction	Andalusia, Spain	[29]

39	Eye blurry and itching	Aerial parts, inhaled into eyes	Balikesir, Turkey	[134]
		Seeds, roots, and leaves	Northern Portugal	[28]
		Seed, infusion, edible	Gujranwala, Pakistan	[132]
		Leaves and/or fruits	South Africa	[27]

40	Cough	Whole plant, oral infusion	Guerrero, Mexico	[44]
		Whole plant, decoction	Southern Spain	[29]
		Whole plant	Western cape of South Africa	[135]

41	Stomachache	Whole plant, oral infusion	Guerrero, Mexico	[44]
		Fruit	Middle Navarra	[130]
		Seed decoction is used against stomach ache	Liguria, Italy	[45]
		Seed, leaf, and stem-infusion, edible	North Iran	[24]

42	Stress removal	Apical shoots is used as sedative for children	Liguria, Italy	[45]
			Southern Punjab, Pakistan	[25]

43	Flatulence	Leaf and fruit, infusion	Brazil	[133]
		Leaf and seeds, infusion	Northern Badia, Jordan,	[39]
		Fresh fruit, decoction	North Bengal, India	[38]

**Table 6:** Volatile compounds present in essential oil of *Foeniculum vulgare*.

Sr. number	Compounds
1	*α*-Thujene
2	1,8-Cineol
3	*β*-Ocimene
4	Linalool
5	Germacrene D
6	Anisketone
7	Apiol
8	*n*-Hexadecanoic acid
9	Cubebene
10	Benzene-1-methyl-4-(1-methylethyl)-*p*-cymene
11	1,3,6-Octatriene, 3,7-dimethyl-, (E)-3-carene
12	2-Heptene
13	3-Methyl-butanal
14	*β*-Pinene
15	Camphene
16	Hexanal
17	*α*-Pinene
18	*β*-Phellandrene
19	*α*-Phellanrrene
20	*β*-Myrcene
21	4-Carene
22	2-Heptanohe
23	Limonene
24	4-Methyl-bicyclo[3.1.0]hex-2-ene
25	Eucalyptol
26	*α*-Pinene
27	*γ*-Terpinene
28	7-Dimethyl-1,3,7-octriene
29	2,4-Dimethyl-benzenamine
30	3-Carene
31	Cathine
32	2-Heptanol
33	2-Propyn-1-ol
34	2,6-Dimethyl-2,4,6-octatriene
35	Fenchone
36	1-Methyl-4-(1-methylethyl)-benzene
37	*cis*-Limonene oxide
38	*trans*-Limonene oxide
39	6-Methylene-bicyclo[3.1.0]hexane
40	Sabinene hydrate
41	Fenchyl acetate
42	Camphor
43	Benzaldehyde
44	1,3-Butanediol
45	Dicyclopropyl carbinol
46	Fenchol
47	1-Octanol
48	5-Methyl-2-heptanol
49	Tetradecyl-oxirane
50	Estragole
51	*Trans*-*p*-2,8-menthadien-1-ol
52	*β*-Terpinol
53	*cis*-*p*-2,8-Menthadien
54	4-Methyl-1-(methylethyl)-3-cyclohexen
55	2-Methyl-5-(1-methylethyl)-2-cyclohexen-1-one
56	Phenylmethyl-formic ester
57	2,3-Cyclohexen-1-methanol
58	* Epi*-bicyclosesquiphellardrene
59	*cis*-*p*-Menth-2,8-dienol
60	1,4-Dimethoxy-benzene
61	1-Methoxy-4-(1-propenyl)-benzene
62	1,2,4a,5,8,8a-Hexadehyde-naphthalene
63	4-Methyl-bicyclo[3.1.1]hept-3-en-2-ol
64	*trans*-Anethole 73.20 73.27 66.71
65	Allantoic acid
66	2-Methyl-5-(1-methylethyl)-phenol
67	Mannoheptulose
68	2-Methyl-5-(1-methylethyl)-2-cyclohexen-1-ol
69	1-Undecanol
70	Benzothiazole
71	E-Pinane
72	2-Cyclohexen-1-ol
73	2-Methyl-bezenemethanol
74	4-Methoxy-benzaldehyde
75	1,6-Hexanediol
76	2-Methoxycyclohexanone
77	*β*-Elemenone
78	Mephenesin
79	4′-Methoxy-acetophenone
80	2-Methyl-3-methylethyl-butanoic acid
81	Folic acid
82	1-(Methoxyphenyl)-2-propanone
83	1-Methyl-3-(1-methylethyl)-benzene
84	4-Fluorohistamine
85	1,2-Dimethoxy-4-(1-propenyl)-benzene
86	(E)-2-Hydroxy-4-cyano-stilbene
87	1-(3-Methoxyphenyl)-1-propanone

**Table 7:** Biological activities of some phytoconstituents reported in different parts of *Foeniculum vulgare*.

Sr. number	Biological activities	Part used^a^	Phytochemicals	Reference
1	Oestrogenic	SDEO	Dianethole, photoanethole	[71]

2	Hepatoprotective	SDEO	*β*-Myrcene, Limonene	[9]

3	Antithrombotic	SDEO	*trans*-Anethole	[55]

4	Human liver cytochrome P450-3A4 inhibitory	SD	5-Methoxypsoralen	[83]

5	Antiradical scavenging	FW	3-Caffeoylquinic acid, quercetin-3-O-galactoside, kaempferol-3-O-glucoside, kaempferol-3-O-rutinoside, rosmarinic acid	[67]
		AP	3,4-Dihydroxyphenethyl-alchohol-6-O-caffeoyl-*β*-D-glucopyranoside, 3′,8′-binaringenin	[70]

6	Antioxidant	FT	*cis*-Miyabenol C	[139]

7	Anticancer	SDEO	Anethole	[110]

8	Antibacterial	ST	Dillapiol, psoralen, bergapten, scopoletin, imperatorin, dillapional,	[100]

9	Antimycobacterial	ST, LF	2,4-Undecadienal, linoleic acid, oleic acid, 1,3-benzenediol, undecanal	[63]

10	Repellent	FT	(z)-9-Octadecanoic acid, fenchone	[117]

11	Acaricidal	SDEO	*para*-Anisaldehyde	[118]

12	Insecticidal	SDEO	1,8-Cineole, terpineol	[120]

^a^AP: aerial part, FT: fruit, LF: leaf, SD: seed, SDEO: seed essential oil, ST: stem, and FW: fennel waste.

**Table 8:** Details of pharmacological/biological activities reported from *Foeniculum vulgare*.

Activity	Plant part used	Dosage form/type of extract	Concentration/dosages	Tested living system/organ/cell/type of study	Results	References
Antiinflammatory	Fruit	Methanolic Extract	200 mg/kg: oral administration	*Invivo,* male ICR mice, BALB/c mice, and Sprague-Dawley rats	Inhibitory effects against acute and subacute inflammatory diseases and type IV allergic reactions	[79]

Hepatoprotective	Seed	Essential oil	0.4 mL/kg	*Invivo,* carbon tetrachloride induced liver injury model in male Sprague-Dawley rats	Decreases the level of serum enzymes, namely, aspartate aminotransferase (AST), alanine aminotransferase (ALT), alkaline phosphatase (ALP), and bilirubin	[9]

Hypoglycaemic	Seed	Essential oil	30 mg/kg	*Invivo,* streptozotocin induced diabetic rats	Ingestion of essential oil to diabetic rats corrected the hyperglycemia and the activity of serum glutathione peroxidase and also improved the pathological changes noticed in their kidney and pancreas	[12]

Antihirsutism	Seed	Fennel extract	Creams containing 1%, 2% of fennel extract and placebo	45 female patients aged 16–53 years with mild to moderate forms of idiopathic hirsutism	Cream containing 2% fennel is better than the cream containing 1% fennel and these two were more potent than placebo	[78]

Cytoprotective	Fruit	Methanolic extract	200 *μ*g/mL	Normal human blood lymphocyte	Provides more cytoprotection for normal human lymphocytes as compared with standard sample, that is, doxorubicin	[11]

Antitumor	Fruit	Methanolic extract	25 to 200 *μ*g/mL	B16F10 melanoma cell line	70% methanolic extract shows good antitumour activity at the concentration of 200 *μ*g/mL.	[11]

Antioxidant	Seed	Ethanol and water extract	100 *μ*g of ethanol and water extract	*Invitro*, not stated	77.5% and 99.1% inhibition of peroxidation in linoleic acid system, respectively.	[10]

Oestrogenic	Seed	Acetone extract	Not stated	*Invivo,* female rats	Weight of mammary glands increases also increases the weight of oviduct, endometrium, myometrium, cervix, and vagina	[8]

Vascular effects	Leaf	Aqueous extracts	0.1 to 0.4 mL injection	*Invivo,* pentobarbital-anaesthetised Sprague-Dawley rats	Significant dose-related reduction in arterial blood pressure, without affecting the heart rate or respiratory rate	[75]

Antistress	Fruit	Aqueous extracts	50, 100 and 200 mg/kg	*Invivo,* scopolamine-induced amnesic rats	Significant inhibition of the stress induced biochemical changes in vanillyl mandelic acid and ascorbic acid.	[13]

Memory-enhancing	Fruit	Aqueous extracts	50, 100, and 200 mg/kg	*Invivo,* scopolamine-induced amnesic rats	The significant reduction is achieved in amnesia in extract-treated groups as compared with the control group of animals	[13]

Chemopreventive	Seed	Test diet of fennel	4% and 6% test diets of Fennel	*In-vivo,* DMBA-induced skin and B(a)P-induced forestomach papillomagenesis in Swiss albino mice	Significant reduction in the skin and the forestomach tumor incidence and tumor multiplicity as compared to the control group of animal	[85]

Oculohypotensive	Seed	Aqueous extract	0.3%, 0.6%, and 1.2% (w/v)	*Invivo,* rabbits	It exhibits 17.49, 21.16, and 22.03% reduction of intraocular pressure (IOP) in normotensive rabbits at 0.3%, 0.6%, and 1.2% (w/v) concentrations of extract	[84]

Anticarcinogenic	Seed	Methanolic extract	100 mg/kg	*Invivo,* Swiss albino mice	Significant increase in malondialdehyde levels and the significant decrease in catalase activity and glutathione content in liver and tumor tissue in mice bearing Ehrlich ascites carcinoma	[140]

Antiaging	Seed	Fennel extract	Formulation containing 4% extract	Male volunteers with mean age of 48 years	Formulation showed significant effects on skin moisture and transepidermal water loss	[50]

Apoptotic	Fruit	Ethanol extract	100 to 300 *μ*g/mL	Nine human cell lines: ML-1, J-45.01, HL-60, 1301, U-266B1, WICL, C-8166, EOL, and H-9—human T cell	Highest mortality in Trypan blue test for J45 cell line, 4% of viable cells and for C8166 cell line, 100% of mortality	[86]

Antiulcerogenic	Aerial parts	Aqueous extract	75, 150, 300 mg/kg	*Invivo*, ethanol induced gastric lesions in Sprague-Dawley rats	Pretreatment with extracts significantly reduced ethanol induced gastric damage.	[81]

Cytotoxic	Root (ground part)	Dichloromethane and methanol (1 : 1) extract	700 *μ*g/mL	Murine fibrosarcoma L929sA cells and on the human breast cancer cells MDA-MB231 and MCF7	Cytotoxic activity may act via inhibition of the NFkB pathway.	[82]

Antimycobacterial	Aerial parts	Chloroform, hexane, methanol, and aqueous extracts	100 to 200 *μ*g/mL	*Invitro*, *M. tuberculosis *H37Rv (27294)	Hexane extract is active against pan sensitive strain of *M. tuberculosis *H37RV	[141]

**Table 9:** Antibacterial, antimycobacterial, antifungal, and antiviral studies carried out on *Foeniculum vulgare*.

Sr. number	Part used^a^	Type of extract	Active strains^b^	Method	Reference standard	Effective concentration	Reference
1	SD	Essential oil	*S.a.*, *Enterococcus *sp., *P.a.*, *E.c.,* and *Salmonella *sp.	Filter paper disc diffusion method	0.5 Mac Farland's Standard (1.5 × 108 CFU/mL)	10 *μ*L/disk	[142]

2	FT	Essential oil	*E.c., B.m.,*and 27 phytopathogenic bacterial species	Agar diffusion method	Rifampicin	1.6 mg/mL	[143]

3	AP	Aqueous, ethanol and ethyl-acetate extracts	*A.r.t.*, *Er.c.*,* P.f.,*and *P.g. *	Filter paper disc diffusion method	Chloramphenicol, streptomycin, and tetracycline	15 mg per disc.	[91]

4	SD	Essential oil	*E.a.*, *S.t.*, *S.a.*, *St.e.*, *E.c.*, *P.a.,*and *C.a. *	Filter paper disc diffusion method	Amoxicillin and cefazolin	15 *μ*L/disk	[144]

5	FL, FT	Essential oil	*A.a.*, *F.o.,* and *R.s. *	Filter paper disc diffusion method	NS	10 and 40 ppm	[95]

6	FL, LF, TW	Essential oil	*Bacilli *sp., *P.a.*, *Acinetobacter *sp., and *A.f. *	Agar diffusion method	Fleroxacin	30, 25, 20, 15 and 10 *μ*L per well	[145]

7	SD, ST, LF, RT	Essential oil	*S.a.*, *B.s.*, *E.c.*, *P.a.*, *C.a., C.t., M.s.*, *M.c.,* and *M.x. *	Agar dilution method	NS	NM	[146]

8	SD	Essential oil	*E.c.*, *B.s.*, *A.n.*, *F.s.,*and *Rh.s. *	Filter paper disc diffusion method	Amoxycillin and flumequine	300 *μ*g/disc	[96]

9	FT	Essential oil and ethanolic and methanolic extracts	*B.c.*, *B.m.*, *B.p.*, *B.s.*, *E.c.*, *K.p.*, *M.l.*, *P.p.*, *P.s.*, and *C.a. *	Filter paper disc diffusion method	Cefoperazone, sulbactam, ofloxacin, and netilmicin	30 mg/mL	[92]

10	SD	Aqueous/organic extracts	*E.f.*, *S.a.*, *E.c.*, *K.p.*, *P.a.*, *Sa.t.*, *S.t.,* and *S.f. *	Agar well and disc diffusion method	Chloramphenicol, gentamicin, and ampicillin	NM	[3]

11	SD	Essential oil	*E.c.*, *P.a.*, *S.a.*, *B.s.*, *A.n.,* and *C.a. *	Filter paper disc diffusion technique	Ampicillin and miconazole nitrate	10 *μ*L/disk	[113]

12	SD	Ethanol, methanol, and aqueous extracts	*E.c.*, *K.p.*, *P.v.*, *E.a., Sa.t., B.c.,*and *S.a. *	Agar well and disc diffusion method	Streptomycin	NM	[4]

13	SD	Essential oil	*E.c., P.a., S.a.*,* C.a.,*and* A.n. *	Cylinder-plate diffusion method	NS	0.25 to 2.0%	[147]

14	FT	Essential oils	*S.a.*, *B.c.*, *P.a.*, *E.c.,*and *C.a. *	Disc paper and broth microdilution methods	NS	NM	[148]

15	SD	Methanol, ethanol, diethyl ether, and hexane extract	*E.c.*, *Sa.t.*, *B.c.*, *S.a.*, *C.a.,*and *As.f. *	Filter paper disc diffusion technique	NS	7.5, 10, 12.5, 15, 20 *μ*g/disk	[23]

16	LF, FL	Crude, chloroform, and methanol extract	*E.c. *and *S.a. *	Filter paper disc diffusion method	NS	NM	[93]

17	FT	Essential oil	HSV-1 and PI-3	Using Madin-Darby bovine kidney and Vero cell lines	Acyclovir	0.025 to 0.8 *μ*g/mL	[5]

18	LF	Essential oil	*S.a.*, *E.c.*, *K.p.*, *P.a.*, *S.e.*, *C.a.* and P.m., *A.n.,*and *F.o. *	Filter paper disc diffusion method	Gentamicin, amoxicillin, and nystatin	5 *μ*L/disk	[94]

19	ST, LF	Hexane extract	*M.t. *	96-well sterile microtiter plate assay	NS	200 *μ*g/mL	[63]

20	SD	Essential oil	*S.a., E.c., K.p.,*and* P.a. *	Agar well diffusion method	Imipenem	50 *μ*L/well	[149]

21	SD	Essential oil	*S.a., E.c., S.c.,*and *St.f. *	Filter paper disc diffusion method	Amoxicillin	10, 50, 100 *μ*L/mL	[150]

22	SD	Essential oil	*S.a., B.s., B.m., B.c., S.l., S.h., Sa.t., S.d., S.s., Sh.s., S.b., E.c.,*and *P.a. *	Filter paper disc diffusion method	Streptomycin	1 *μ*g/mL	[151]

23	FT	Essential oil	*C.a. *	Agar well and filter paper disc diffusion method	Fluconazole and nystatin	25 *μ*L/well and 15 *μ*L/disc	[152]

24	SD	Methanolic extract	*E.c.*, *P.a.*, *S.a.,* and* B.p. *	Agar diffusion method	Chloramphenicol and ampicillin	NM	[7]

25	SD	Aqueous and alcoholic extracts	*A.a.*, *M.r.,*and *A.f. *	Agar well diffusion method	NS	NM	[98]

^a^AP: aerial part, FL: flower, FT: fruit, LF: leaf, RT: root, SD: seed, ST: stem, and TW: twig.

^b^ *A*.*a.: Alternaria alternate, A.f.: Alcaligenes faecalis, As.f.: Aspergillus flavus, A.n.: Aspergillus niger, A.r.t.: Agrobacterium radiobacter pv. tumefaciens, B.c.: Bacillus cereus, B.m.: Bacillus megaterium, B.p.: Bacillus pumilus, B.s.: Bacillus subtilis, C.a.: Candida albicans, C.t.: Candida tropicalis, E.a.: Enterobacter aerogenes, Er.c.: Erwinia carotovora, E.c.: Escherichia coli, E.f.: Enterococcus faecalis, F.o.: Fusarium oxysporum, F.s.: Fusarium solani, K.p.: Klebsiella pneumonia, M.c.: Mycobacterium chelonae, M.l.: Micrococcus luteus, M.r.: Mucor rouxii, M.s.: Mycobacterium smegmatis, M.t.: Mycobacterium tuberculosis H37Rv ATCC 27294, M.x.: Mycobacterium xenopi, P.a.: Pseudomona aeruginosa, P.f.: Pseudomonas fluorescens, P.g.: Pseudomonas glycinea, P.m.: Phytopathogenic molds, P.p.: Pseudomonas putida, P.s.: Pseudomonas syringae, P.v.: Proteus vulgaris, R.s.: Rhizoctonia solani, Rh.s.: Rhizopus solani, S.a.: Staphylococcus aureus, S.b.: Shigella boydii, S.c.: Staphylococcus coagulase, S.d.: Shigella dysenteriae, S.e.: Salmonella enteritidis, S.e.: Staphylococcus epidermidis, S.f.: Shigella flexneri, St.f.: Streptococcus faecalis, S.h.: Streptococcus haemolyticus, S.l.: Sarcina lutea, S.s.: Shigella shiga, S.t.: Salmonella typhimurium, Sa.t.: Salmonella typhi, and Sh.s.: Shigella sonnei*. HSV-1: herpes simplex virus 1 as a representative of DNA viruses and PI-3: parainfluenza-3 virus (PI-3) as representative of RNA viruses.

NS: no reference standard employed and NM: not mentioned.
